# Photodynamic Inactivation as a New Weapon Against Plant Fire Blight Disease: Proof of a New Dawn of Environmentally Friendly Crop Protection

**DOI:** 10.1021/acs.jafc.5c02074

**Published:** 2025-08-01

**Authors:** Mariana Vasconcelos, Ying Piao, Sebastian Himbert, Andreas Fellner, Fengyan Wang, Jun Liu, Michael Fefer, Youqing Shen, Maikel C. Rheinstädter, George W. Sundin, Kristjan Plaetzer

**Affiliations:** a Laboratory of Photodynamic Inactivation of Microorganisms, Department of Biosciences and Medical Biology, University of Salzburg, Hellbrunner Str. 34, Salzburg 5020, Austria; b Zhejiang Key Laboratory of Smart BioMaterials and Center for Bionanoengineering, College of Chemical and Biological Engineering, 12377 Zhejiang University, Hangzhou 310027, China; c Department of Physics and Astronomy, 3710 McMaster University, Hamilton, Ontario L8S 4M1, Canada; d Department of Chemical Engineering, 3710 McMaster University, Hamilton, Ontario L8S 4M1, Canada; e Suncor Energy Inc, 150-6 Avenue SW, Calgary, Alberta T2P 3E3, Canada; f Department of Plant, Soil and Microbial Sciences, 3078 Michigan State University, 578 Wilson Rd., East Lansing, Michigan 48824, United States

**Keywords:** *Erwinia amylovora*, blossom blight, apple

## Abstract

Fire blight, caused by , severely affects apple and pear orchards on a global scale. Conventional treatment includes the application of antibiotics such as streptomycin during bloom, which promotes resistance. Photodynamic inactivation (PDI) is based on the light-induced and photosensitizer-mediated overproduction of reactive oxygen species for killing microbes. This study develops PDI using the natural photosensitizer sodium magnesium chlorophyllin (Chl) from the laboratory to field application. Laboratory experiments showed commercial-ready SUN-D products (Chl + EDTA) were highly effective against (395 nm LED, total kill at 53.2 J cm^–2^), regardless of streptomycin resistance phenotype. No resistance to Chl developed after 15 treatment cycles. Molecular dynamics simulations suggest that EDTA lowers the energy barrier for chlorin e6, enhancing membrane penetration. Field trials at two locations using sunlight illumination showed that SUN-D controlled blossom blight comparably to antibiotics. Photodynamic inactivation with SUN-D offers a promising, resistance-independent addition to current fire blight management strategies.

## Graphical abstract

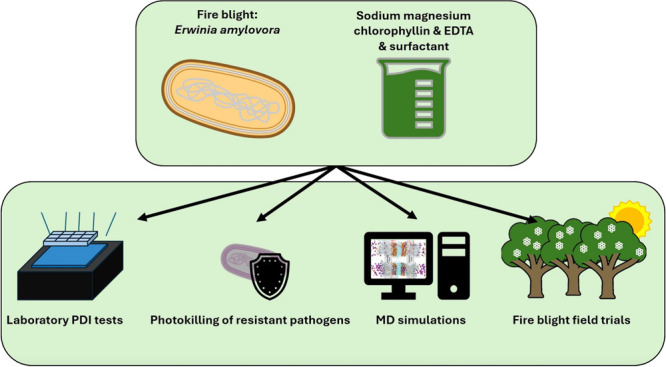

## 1 Introduction

Fire blight, caused by the bacterial pathogen , is a devastating disease and is a critical limiting factor to commercial pome fruit production. Fire blight was first described in North America in 1790 and has since spread globally into most countries with commercial pome fruit production, with most recent incursions into central Asia, Korea, and China.^1–3^ The significance of fire blight includes both current season yield loss due to flower infections and systemic infections, leading to rootstock blight and tree death. is capable of infecting flowers, fruits, vegetative shoots, woody tissues, and rootstock crowns, leading to blossom, shoot, and rootstock blight symptoms.^4^

Blossom blight disease symptoms (flower death) of fire blight are typically initiated in the spring on flowers as overwintering inoculum from cankers is disseminated to the surface of stigmas by rain and insects.^5^ The surface of the tips of stigmas particularly favors the exponential growth of in the intercellular spaces between papillae cells.^6,7^ Under conducive weather conditions, populations of 10^6^ to 10^7^ cells per flower are common.^8^ The availability of free moisture through rain or heavy dew is required to enable cells to migrate down the style, where these cells infect flowers through natural openings present in the nectaries.^9^ Within a few days after blossom blight symptoms are present, the emergence of ooze poses a critical problem, as ooze contains extremely high numbers of cells^10^ that are readily spread by wind and rain, and these cells can be disseminated to shoot tips, causing new infections and the shoot blight phase of fire blight.

Management of blossom blight is critically important in apple and pear orchards, both to preserve current season yield, to prevent systemic infection of trees, and to prevent the massive buildup of inocula that can cause epidemics of shoot blight infection in orchards. For over 50 years (ca. 1960–2010s), United States apple growers relied on the bactericidal antibiotic streptomycin (Sm) for fire blight management during bloom.^11^ Unfortunately, a continued reliance on streptomycin led to the periodic evolution of streptomycin resistance in , which was first detected in the Pacific Northwest in the 1970s, with subsequent detections in California, Michigan, and New York.^12–16^ In several cases, streptomycin resistance was conferred by the *strA-strB* genes that are transferable within pathogen populations.^17,18^ The antibiotics oxytetracycline and kasugamycin have been utilized as replacements for streptomycin in orchards with streptomycin resistance; however, these antibiotics are also at risk of resistance evolution, and oxytetracycline resistance has recently been reported in a California population of .^19^

Alternatives to antibiotics for blossom blight management have been widely investigated over the years. In recent field studies, the efficacy of some copper products was good and similar to antibiotic standards^20,21^ but copper, when applied at bloom, can cause fruit russeting on some apple cultivars. In addition, the biological control Blossom Protect, which consists of the yeast and an acidifying buffer, has performed similarly to antibiotic standards in multiple field trials across the United States.^21^ A recent study has shown that Blossom Protect induces a systemic acquired resistance response in apple flowers that likely contributes to the positive results of field efficacy for blossom blight.^22^ In contrast, recent examinations of potential biological controls and biorational products with field studies conducted at multiple locations have shown that materials such as *Bacillus* spp. fermentation products, bacteriophages, essential oils, and peracetic acid-peroxide have typically yielded moderate disease suppression compared to antibiotic controls, with some variability in results observed between years.^21,23^ Thus, there is a critical need for novel efficacious alternatives to antibiotics for fire blight management.

Photodynamic inactivation (PDI) of microorganisms is a derivative of photodynamic therapy (PDT), an established clinical modality for eliminating undesirable or pathogenic cells in various malignant and nonmalignant conditions.^24^ Mechanistically, both PDI and PDT operate through the same biphasic process: initially, a light-responsive molecule known as a photosensitizer (PS) is absorbed by or bound to the target cells. Subsequently, upon illumination with light of suitable wavelengths, the PS is activated, leading to the excessive generation of reactive oxygen species (ROS), which initiate oxidative processes resulting in cell death. The photophysical and photochemical mechanisms underlying PDI are thoroughly understood: absorption of a photon induces the transition of the PS to its excited singlet state. Photosensitizers exhibit a high probability of intersystem crossing, transitioning to the long-lived excited triplet state. Within this state, the PS shows a high quantum yield for either electron transfer to molecular oxygen (type I photochemical reaction), yielding radicals, or energy transfer, resulting in singlet oxygen generation (type II photochemical reaction). Both types of ROS actively engage in biomolecular oxidation, ultimately culminating in fatal damage to the target cells.^25^

One advantageous aspect is that, unlike antibiotics, PDI exhibits efficacy against a broad spectrum of microorganisms encompassing bacteria, fungi, viruses, and insects,^26–29^ and microbial strains resistant to conventional treatment are most likely susceptible to PDI.^30^ Additionally, owing to its nonspecific mode of action, the development of resistance to PDI is highly improbable.

A wide variety of PS structures exist, and constantly, new photoactive compounds with optimized properties are developed. Most of the currently applied PS is derived from the family of porphyrins, chlorins, and bacteriochlorins featuring strong absorption in the Soret band (410–420 nm) and also in the red to near-infrared part of the electromagnetic spectrum. Phthalocyanines and structures from the BODIPY family are also activated with red or near-infrared light, which is beneficial for penetration in the optical window of human skin.^31^ However, synthesis of these molecules can be extensive, resulting in higher costs of production of these photosensitizers.

For application in agriculture, where very large areas are treated, and large quantities of products are being applied in the environment, the photoactive compounds ideally fulfill the following criteria: (i) availability in large amounts either from natural sources or simple synthesis, (ii) ecofriendliness and high biocompatibility, as well as (iii) rapid photodestruction into harmless products for rapid clearance from ecosystems.

Natural photosensitizers such as hypericin, derived from St. John’s wort, curcumin, extracted from roots of turmeric plants, and chlorophyllin, obtained from simple modification of natural chlorophyll, fulfill most if not all of these criteria.^32,33^ While hypericin and curcumin are poorly soluble in water and therefore require formulation with solubility enhancers such as polyvinylpyrrolidone to be applicable against microorganisms in biological media,^34,35^ sodium magnesium chlorophyllin (Chl) features excellent water-solubility, thus allowing for application against bacteria and fungi in water-based media without additives.^26,27^ It also shows rapid photodestruction, and its biocompatibility is granted by approval as a food colorant (E140 (ii)).^36^

The major component of chlorophyllin is chlorin e6 (Ce6). As an anionic molecule, it is difficult for Ce6 to penetrate the negatively charged outer membrane of Gram-negative bacteria,^30^ which inhibits its application against these microorganisms. Permeating agents, such as the ethylenediaminetetraacetic acid (EDTA) presented here, have been previously demonstrated to overcome these limitations.^26^ While it is well-established that EDTA destabilizes the outer membrane by chelating divalent cations that bridge lipopolysaccharide (LPS) structures,^30,37^ the precise biophysical mechanism by which this disruption facilitates the translocation of specific photosensitizers like Ce6 across the LPS layer has not been fully characterized. In this study, we present a mechanistic molecular-level study demonstrating how EDTA specifically lowers the energetic barrier for the entry of Ce6 into the membrane using MD simulations.

Here, we present a novel, affordable, eco-friendly, and effective formulation of sodium magnesium chlorophyllin for agricultural applications. These commercial-ready SUN-D formulations (Nutrien Ag Solutions) comprise the technical grade Chl, which is more economical than pure Ce6 with disodium EDTA as a surfactant. We evaluated the photokilling efficacy of the SUN-D formulations in both *in vitro* lab assays and *in vivo* field trials on the control of apple fire blight disease (). In addition, to understand the mode of action further, we evaluated the cellular uptake of Ce6 with and without EDTA and used Molecular Dynamics (MD) simulations to investigate the interaction of EDTA with Ce6 in a Gram-negative bacteria mimic.

## 2 Materials and Methods

### 2.1 Bacterial Culture

#### *In* *Vitro* and *In Vivo* PDI Using SUN-D Formulations and Chl

In the in vitro assays, we used the bacterial cell lines ^*WT*^ (SM 30165) and ^*SmR*^,^30^ wild-type and streptomycin-resistant, respectively. The cultures were kept stored at −196 °C in cryovials in a liquid nitrogen freezer. To prepare a fresh bacterial culture, 20 mL of Luria–Bertani (LB) medium (Sigma-Aldrich; St. Louis, MO, U.S.) or LB supplemented with 100 μg/mL streptomycin (Sm) (Sigma-Aldrich) were pipetted into a 100 mL cell culture flask and put on a shaking incubator (ThermoScientific MaxQ 4000, Thermo Fischer Scientific, Waltham, Massachusetts, U.S.), at 200 rpm and 26 °C. After 1 day, 100 μL of the cell culture was pipetted into a new cell culture flask with 20 mL of fresh LB/LB + Sm medium and incubated again under the same conditions. This procedure ensured less than a 1 log step difference in the initial bacterial density for each experimental replicate.

The virulent strain Ea110 ^38^ was used for *in vivo* field testing of the SUN-D formulations. The cell suspensions (1 × 10^6^ CFU ml^–1^) were inoculated to apple trees at the 90% bloom stage (90% flowers opened) as previously described^39^ for field experiments conducted in Michigan, U.S.

#### Bacterial Strain and Cultivation Used for ROS Generation Assay

 ATCC 49946, kindly provided by the School of Life Sciences of Zhejiang University, was used in the cellular uptake assay and the ROS generation assay. The bacteria were cryopreserved at −80 °C in 50% (v/v) glycerol and streaked on nutrient agar (NA) plates (Sigma-Aldrich, Co., St. Louis, Missouri, USA). The strain was grown in liquid NA at 28 °C while being shaken at 200 rpm for 16–18 h to obtain exponentially growing bacteria.

### 2.2 Light Sources

Table 1 provides an overview of the light sources employed in this study.

**Table 1:** Overview of the Light Sources Employed in This Study^b^

experiment	light source/manufacturer	(dominant) wavelength (range)	intensity	radiant exposure/illumination period
*in vitro* assays on PDI and resistance testing	2 × LED array, 480 diodes, type L-7114UVC Kingbright Electronic Europe, Germany, home-built	395 nm	20–33 mW/cm^2^	35.1–78.9 J/cm^2^
singlet oxygen detection of SUN-D-06 solution	RX30 LED Plant Lighting System, Heliospetra AB, Sweden	380–735 nm	400 μmol/m^2^/s	5–30 min
ROS detection of Ce6 with and without EDTA in *Erwinia amylovora*	Youke Instrument & Equipment Corporation, China	380–780 nm plus extra 640 nm	9 mW/cm^2^	32.4 J/cm^2^
field trials—Michigan, USA	natural sunlight	full spectrum	n.d.	760.8 to 978.3 J/cm^2^ per day^a^
field trial—Oregon, USA	natural sunlight	full spectrum	n.d.	760.8 to 978.3 J/cm^2^ per day^a^

a Daily natural solar irradiance for the Michigan and Oregon USA field trial locations was estimated using the Daily Light Integral daily average available at: https://webgis.coe.clemson.edu/storymaps/light-integral-map/

b Abbreviation: n.d. not determined.

#### *In Vitro* PDI and Resistance Assay Using SUN-D Formulations and Chl

The illumination setup consisted of two 395 nm LED sources, one facing down and held 16 cm on top of another identical LED array facing up. A plate shaker (DSG Titertek, Flow Laboratories, Finland) with a clamp was placed in the back of the illumination sources so that the illuminated 24-well plate with 250 μL of DPBS/PS in each well was held between both LED arrays. Both individual LED arrays are composed of 480 LEDs (diode type L-7114UVC, Kingbright Electronic Europe GmbH, Issum, Germany). The plates were illuminated using a dominant wavelength of 395 nm for the time corresponding to the radiant exposure of 53.2 or 78.9 J/cm^2^.

#### Singlet Oxygen Detection of SUN-D-06 Solution

The light source used in the singlet oxygen detection study was a Heliospectra RX30 LED plant lighting system (Heliospectra AB, Gothenburg, Sweden). The light was facing down to illuminate the plates with wavelengths ranging from 380 to 735 nm for 5 to 30 min at 400 μmol/m^2^/s. The fluorescence of the light-treated solution and the dark controls was then measured by a Tecan M1000 plate reader (Tecan Group Ltd., Männedorf, Switzerland) at the excitation/emission wavelength of 488/525 nm with a gain value of 90 and a z-position of 20,000 μm.

#### ROS Detection of Ce6 with and without EDTA in 

The light source used for these experiments (Youke Instrument & Equipment Corporation, Hefei, China) is equipped with an LED lamp covering the wavelength range from 380 to 780 nm and an extra 640 nm red light. The distance between the light source and sample position was 30 cm, and the maximum irradiance at this position was 9 mW/cm^2^ (32.4 J/cm^2^). The maximum intensity of the light source was used for all the experiments conducted under illumination in this section.

### 2.3 PDI Experiments with Chl and SUN-D Formulations

#### Composition of SUN-D Formulations

Sodium magnesium chlorophyllin was obtained as a powder from Carl Roth GmBH + Co KG (purity ≥ 97%, Karlsruhe, Germany). Stock solutions of 10 mM Chl were dissolved in Dulbecco’s modified phosphate buffer (DPBS, pH 7.4) and stored at −20 °C in the dark until use. SUN-D-01 and SUN-D-06 are two plant protection formulations owned by Nutrien Ag Solutions (1313 Lake Fraser Drive SE, Calgary, AB, Canada), comprising sodium magnesium chlorophyllin, disodium EDTA, and an anionic surfactant as an adjuvant. The composition of the photosensitizer formulations in the different dilutions used is shown in Table 2.

**Table 2:** Formulation Composition of the SUN-D Products Used in This Study

formulation	used dilution in ddH_2_O [%]	Chl [mM]	EDTA [mM]	surfactant	mass percentage surfactant [%]
SUN-D-01	0.12	0.75	0.92	alkylnaphthalenesulfonate (CAS number: 68425–94–5)	0.03
	0.24	1.50	1.82		0.07
	0.35	2.19	2.69		0.10
	0.70	4.38	5.38		0.2
SUN-D-06	0.06	0.12	0.69	alkylsulfosuccinate (CAS number: 577–11–7)	0.03
	0.11	0.22	1.27		0.05
	0.23	0.47	2.66		0.10

To prepare the stock solutions in the lab for *in vitro* tests, the powdered formulations were dissolved in Millipore water (Sartorius, Göttingen, Germany) and distributed into 2 mL Eppendorf tubes. The stock solutions were stored in a light-protected box at −20 °C. For the *in vivo* field experiments, SUN-D powdered formulations were mixed in water at concentrations of 0.06, 0.12, 0.24, and 0.36% (v/v) in the spray tank with a final pH of 6.

#### Particle Size Distribution and Singlet Oxygen Detection of SUN-D-06 Solution

Hydrochloric acid (1 M, 0.1M) and sodium hydroxide (1 M, 0.1M) were purchased from LabChem (Zelienople, NY, US). Singlet oxygen sensor green (SOSG) was purchased from Lumiprobe Corp. (Cockeysville, US). All chemicals were used without further purification. All water used in the experiments was deionized and further purified by an EMD Millipore Milli-Q Advantage A10 System (Thermo Scientific, Waltham, MA, US).

In particle size measurements, 0.011% SUN-D-06 solutions were prepared by dissolving SUN-D-06 powder in water. Particle size measurements were performed with a Malvern NanoSight LM10 instrument (Malvern Panalytical, Malvern, UK) equipped with an LM14C laser with a wavelength of 532 nm and a syringe pump (Harvard Apparatus, Catalog NO. 98–5362, Holliston, US). Samples were injected into the chamber at a flow rate of 0.2 mL/min, captured by a complementary metal-oxide semiconductor (CMOS) camera, and analyzed by NanoSight NTA 3.4 software (Malvern Panalytical, Malvern, UK).

In singlet oxygen detection, 0.011% SUN-D-06 solutions were prepared separately by dissolving the powder in water. 0.5 mM singlet oxygen sensor green (SOSG, Lumiprobe, Cockeysville, US) stock solution was prepared by adding 330 μL of methanol to one SOSG tube. In a typical experiment, 2 μL of SOSG and 198 μL of SUN-D-06 were deposited into one well of a 96-well plate (Greiner Bio-one, item number 655090) and mixed. In the control sample, 2 μL of SOSG was mixed with 198 μL of water. Four solution replicas were prepared and measured. In the light experiments, SUN-D-06 samples with SOSG (light, PS) and SOSG alone (light, no PS) were exposed to LED light (Heliospectra RX30, 380–735 nm, 400 μmol/m^2^/s) illuminating from top for 5, 10, 15, 20, 25, and 30 min, whereas the dark controls of SUN-D-06 with SOSG (no light, PS) and SOSG alone (no light, no PS) were placed in the dark for the same time scales. During the treatment, the plates were covered with lids to avoid evaporation. After the light or dark incubation period, the fluorescence of each solution was measured by a Tecan M1000 pro plate reader (excitation/emission: 488/525 nm).

#### *In Vitro* PDI against ^*WT*^ and ^*SmR*^ Using Chl and SUN-D Formulations

One hundred μL of a previously prepared ^*WT*^ or ^*SmR*^ bacterial suspension was pipetted into 20 mL of fresh LB/+100 μg/mL Sm, respectively, and incubated overnight at 200 rpm and 26 °C. Bacterial density in culture reached 10^9^ CFUs/mL and was still in the exponential growth phase.

Bacteria from the overnight culture were centrifuged (833 relative centrifugal force, 3 min) and resuspended in 1800 μL of PS solution (200, 400, and 800 μM of Chl, SUN-D-01 at 0.35% and 0.7%; SUN-D-06 at 0.23% and 0.11%) or ddH2O for the double negative (control −/−) or light control. For experiments conducted with lower bacterial densities, 2 μL (mean: 1.5 × 10^7^ CFU/mL) of culture was used, while for high-density conditions (Figure 5) 2000 μL culture (mean: 2.72 × 10^9^ CFU/mL) was centrifuged. An overview of the Eppendorf tube preparation is given in Supporting Information and Table S1.

On a 24-well plate, 250 μL triplets of each sample were pipetted in. The 24-well plates were incubated in the dark for 5 or 30 min in a plate shaker (Titertek, Pforzheim, Germany) for gentle agitation. After, the 24-well plate with the double negative control and the photosensitizer dark toxicity control triplets was kept in the dark. The 24-well plate with the light control and photosensitizers was uncovered and illuminated. Afterward, a 1:10 serial dilution in DPBS was performed for each sample up to the dilution of 10^–7^. Then, 50 μL of each dilution step of each sample was plated on LB/+100 μg/mL Sm agar plates. The agar plates were incubated for 2 days in a static incubator (Heraeus incubator B 5042 E, Hanau, Germany) at 26 °C in the dark. After the 2 day incubation time, the colony forming units (CFU) were counted.

#### ^*WT*^ PDI Resistance Assay with SUN-D-06

The procedure for the first PDI treatment on ^*WT*^ is similar to the one in the previous section, with the exception that a radiant exposure of 35.1 J/cm^2^ was applied to ensure a 4 to 5 log step inactivation. For the subsequent treatments, the surviving CFU from the treated sample were collected from the agar plate and suspended in LB. The bacterial density of the culture was approximately 5 × 10^6^ CFU/mL. The optical density measured at a 600 nm wavelength (OD_600_) of the suspension was adjusted to 0.4 (4.86 × 10^6^ cfu/mL), and a new treatment cycle was initiated using the previously treated bacterial suspension as an inoculum. This procedure was repeated for 15 treatment cycles.

#### Data Evaluation

For comparison of the different photosensitizer formulations in the PDI assay, the data are displayed as a matter of the relative inactivation achieved with each treatment. This approach is visually a better way to detect the effectiveness of the products. To obtain the relative inactivation value of each sample, the CFU/100 μL mean of the double negative control, control −/–, was divided by the CFU/100 μL mean of each sample. When a sample showed no CFU (limit of detection), the relative inactivation value would be considered the CFU/100 μL mean of the control −/– sample. The mean and the standard deviation of the relative inactivation of all samples were calculated, and the graphs were plotted using the software OriginPro 2021b (OriginLab, Massachusetts, US). A photosensitizer formulation is effective against bacterial infection when a relative inactivation of three logarithmic steps (3 log) is achieved (killing of 99.9%).^40,41^

### 2.4 Mechanism Study with Ce6 and EDTA

#### Bacteria Uptake of Ce6 with and without EDTA

Chlorin e6 (Ce6) and disodium ethylenediaminetetraacetic acid (EDTA) were purchased from Yuanye Chemical (Shanghai, China). The intracellular uptake of Ce6 by was studied by measuring the fluorescence intensity of Ce6 in the bacteria using flow cytometry (FACS Calibur, BD, Hangzhou, China) at an excitation wavelength of 488 nm and an emission wavelength of 670 nm. The bacteria were adjusted to an OD_600_ of 0.5 (5 × 10^6^ cfu/mL), and then different concentrations of Ce6 (0.2 mM) and EDTA (0.5/2/5 mM) were separately added to reach a final volume of 500 μL in each tube. After incubation for 0.5 or 3 h, the bacterial suspension was centrifuged at 3000*g* and washed with phosphate-buffered saline (PBS) three times. The bacteria were resuspended with 500 μL of PBS (pH 7.2–7.6) and subjected to flow cytometry analysis. Histograms were analyzed using fluorescence correlation spectroscopy (FCS) expressing Ce6, and the mean fluorescence intensities and the positive percentages were recorded.

Confocal laser scanning microscopy (CLSM, Nikon A1) was also employed to visualize the uptake of Ce6. In the Ce6 intracellular uptake study, cells were mixed with Ce6 (0.2 mM) and EDTA (0.5/2/5 mM). After incubation for 0.5/3 h at 28 °C, the cells were washed with PBS and imaged by CLSM (Ex: 640 nm Em: 670–720 nm).

#### ROS Detection of Ce6 with and without EDTA in 

A stock solution of Ce6 (2.0 mM) was prepared by dissolving Ce6 in a NaOH solution at pH 9.0. The solution was stored at 4 °C in the dark. Dihydroethidium (DHE) was used as the fluorescent probe for the detection of ROS generation in the presence of live cells. DHE is typically the detector of type I ROS of superoxide and hydrogen peroxide. Ce6 was commonly considered to induce the production of type II ROS of singlet oxygen (^1^O_2_). It has been shown that the singlet oxygen could be transferred to type I superoxide anions (O^2–^).^42–46^ We found that it is effective to detect ROS generated by Ce6 with a DHE probe in live cells. Briefly, exponentially growing were seeded into each well of a 24-well plate at a density of ∼5 × 10^6^ CFU/mL. Ce6 (40 μM) or Ce6 with EDTA (0.27 mM) was added into each well in a volume of 500 μL. After incubation in the dark for 30 min, the bacteria were then illuminated for 1 h. The ROS probe dihydroethidium (Beyotime Biotechnology S0063, Hangzhou, China) was added to each well and then illuminated for 30 min. The suspension in each well was transferred to separate tubes. The bacteria in each tube were collected by centrifugation and washed with PBS three times. The washed bacteria were then dispersed in 0.2 mL of PBS, and the fluorescence intensity of the bacteria was measured using a fluorescence microplate reader (Molecular Devices, SpectraMax M2, lambda­(Ex) = 360 nm, lambda­(Em) = 610 nm).

#### 2.5 Field Trials: *In Vivo* PDI on apple fire blight

Field tests to evaluate the efficacy of PDI for fire blight disease management were conducted annually at the Michigan State University Plant Pathology farm (MSU-PLP; 42°68′92.80″N, −84°48′47.50″W) between 2020 and 2023. An additional field test was conducted in Junction City, Oregon (44°21′76.13″N, −123°14′11.71″W) in 2023. Estimates of daily solar irradiance (photosynthetically active radiation, 400–700 nm) for both field sites were done using the Daily Light Integral^47^ and taken from monthly averages using high-resolution maps available at: https://webgis.coe.clemson.edu/storymaps/light-integral-map/. The efficacy of the SUN-D-01 and SUN-D-06 test compounds at all rates used was assessed in at least two experiments and compared to that of the antibiotic standards streptomycin (FireWall 17WP; AgroSource, Tequesta, FL) or kasugamycin (Kasumin 2L; UPL, Cary, NC). At the MSU-PLP, a randomized complete block design with four single-tree replicates was used to evaluate the management programs. Treatments were applied at 2,806 L ha^–1^ with a handgun sprayer at 138–172 kPa, and were applied at 75% bloom, full bloom, and petal fall. Trees were inoculated with Ea110 by spraying bacterial suspensions (1 × 10^6^ CFU mL^–1^) at 90% bloom using an 11.3 L hand-pumped backpack sprayer. Blossom blight and shoot blight were evaluated at approximately 1 and 1.5 months, respectively, after the final spray application. Data were analyzed using the analysis of variance and least significant difference (LSD) mean comparison function of ARM (ver. 9.3; Gylling Data Management Inc., Brookings, SD, US). Percentage data were subjected to an arcsine-square root transformation before analyses. At the Oregon site, a similar randomized complete block design and application strategy were used with four three-tree replicates. At the Oregon site, we relied on natural inoculum; thus, no inoculations were done.

## 3 Results

### 3.1 Particle Size and Singlet Oxygen Detection of SUN-D-06 Solution

Multiple peaks were observed for 0.011% SUN-D-06 solutions in the NanoSight particle size measurement, as shown in Figure 1, indicating that particles in solutions are polydispersed. NanoSight results in Table 3 showed that particles had a mean hydrodynamic diameter of 145 nm. Singlet oxygen sensor green (SOSG) is a specific singlet oxygen detector that reacts with it to generate strong green fluorescence. Solutions’ fluorescence intensity increased as a function of time after being exposed to light, indicating singlet oxygen was produced in solutions. The mixture of SUN-D-06 and SOSG after light exposure showed significantly higher fluorescence intensity than the same solutions in the dark, suggesting that Chl in SUN-D-06 acts as a photosensitizer to produce singlet oxygen (Figure 2). After 30 min of radiant exposure, the fluorescence intensity of SOSG alone showed an evident slight increase, which was consistent with the previous report that SOSG can generate singlet oxygen under light exposure.^48^

**Figure 1:**
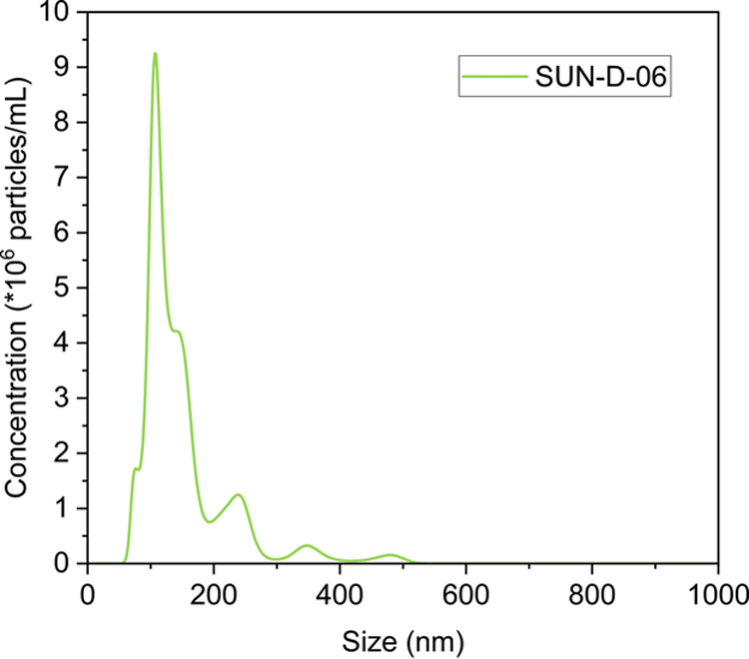
NanoSight particle size distribution of the 0.011% SUN-D-06 solution.

**Figure 2:**
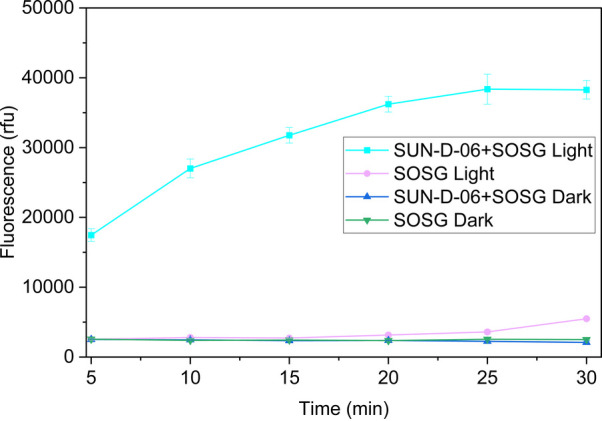
Fluorescence intensity (excitation/emission wavelength: 488 nm/525 nm) of singlet oxygen sensor green (SOSG) and SUN-D-06 with and without illumination with a Heliospectra RX30 LED light (380–735 nm) at 400 μmol/m^2^/s. The higher fluorescence intensity indicates more singlet oxygen produced. Error bars reflect the standard error of the mean from four independent measurements.

**Table 3:** Particle Size (nm) and Particle Concentration (×10^8^particles/mL) in 0.011% Sun-D-06 Solution Measured by NanoSight Particle Analyzer

nanoparticle analysis in 0.011% Sun-D-06	
mean ± SD (nm)	145 ± 12
concentration (×10^8^ particles/mL)	5.7 ± 1.5

### 3.2 *In Vitro* PDI against ^*WT*^ and ^*SmR*^

The toxicity of 200–800 μM Chl without additives on ^*WT*^ cultures in LB was assessed. No significant Chl phototoxicity to ^*WT*^ was observed. Figure 3 shows that even the samples treated with 800 μM Chl and subjected to a radiant exposure of 78.9 J/cm^2^ exhibited relative inactivation levels that remained below the antibacterial effect of three log steps (99.9% reduction).

**Figure 3:**
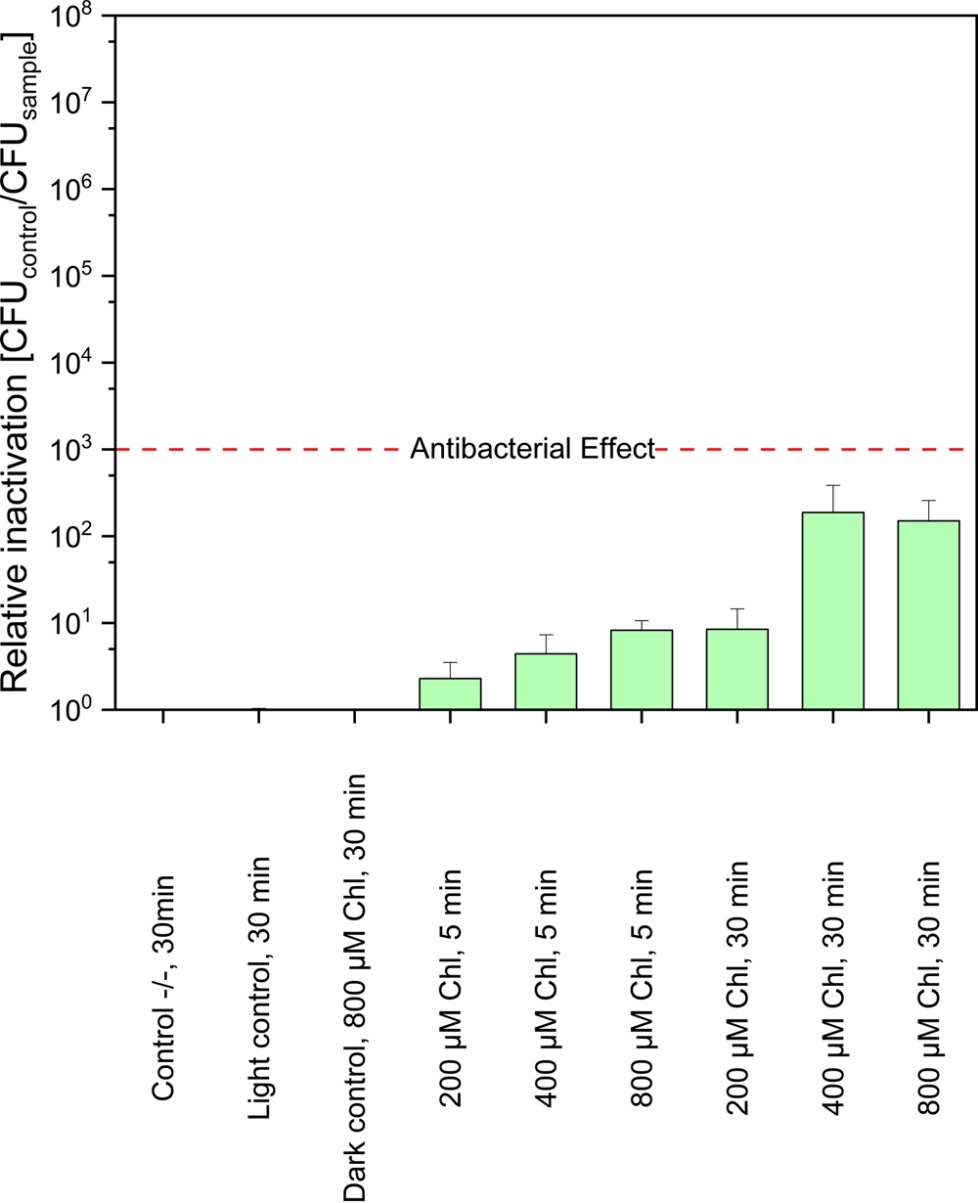
Photodynamic Inactivation based on chlorophyllin without additives shows low toxicity toward ^WT^ cultured in LB medium. Each bar reports results from three replicate samples. Dark incubation: 250 μL, for 5 and 30 min, double-sided illumination, 395 nm. Radiant exposure: 78.9 J cm^–2^. Control −/–: double negative control, light control: light only control, no photosensitizer. Number of CFU on control −/–: 8.36 × 10^6^/mL. The red dashed line represents an antibacterial effect, corresponding to a reduction of 3 log units. Error bars denote standard deviation. Relative inactivation is CFU control −/– divided by the CFU sample.

Next, the photokilling performance of SUN-D-01 and SUN-D-06, formulations comprised of Chl and EDTA, was assessed. The efficacy of PDI using these photosensitizer products was evaluated both on the bacterial wild-type and streptomycin-resistant strains, ^*WT*^ and ^*SmR*^ *,* respectively. The effectiveness of SUN-D-01 was analyzed both at the standard concentration of 0.35% and at the doubled concentration of 0.7%. The incubation times were 5 and 30 min, and the radiant exposure was 53.2 J/cm^2^. As can be observed in Figure 4, PDI employing SUN-D-01 shows low toxicity of the compounds in the dark as well as no light toxicity. Upon illumination, a relative inactivation of at least five log steps can be achieved with a 5 min incubation period. After 30 min of incubation with twice the normal SUN-D-01 concentration (0.7%), a total kill can be achieved with both the resistant and wild-type strain.

**Figure 4:**
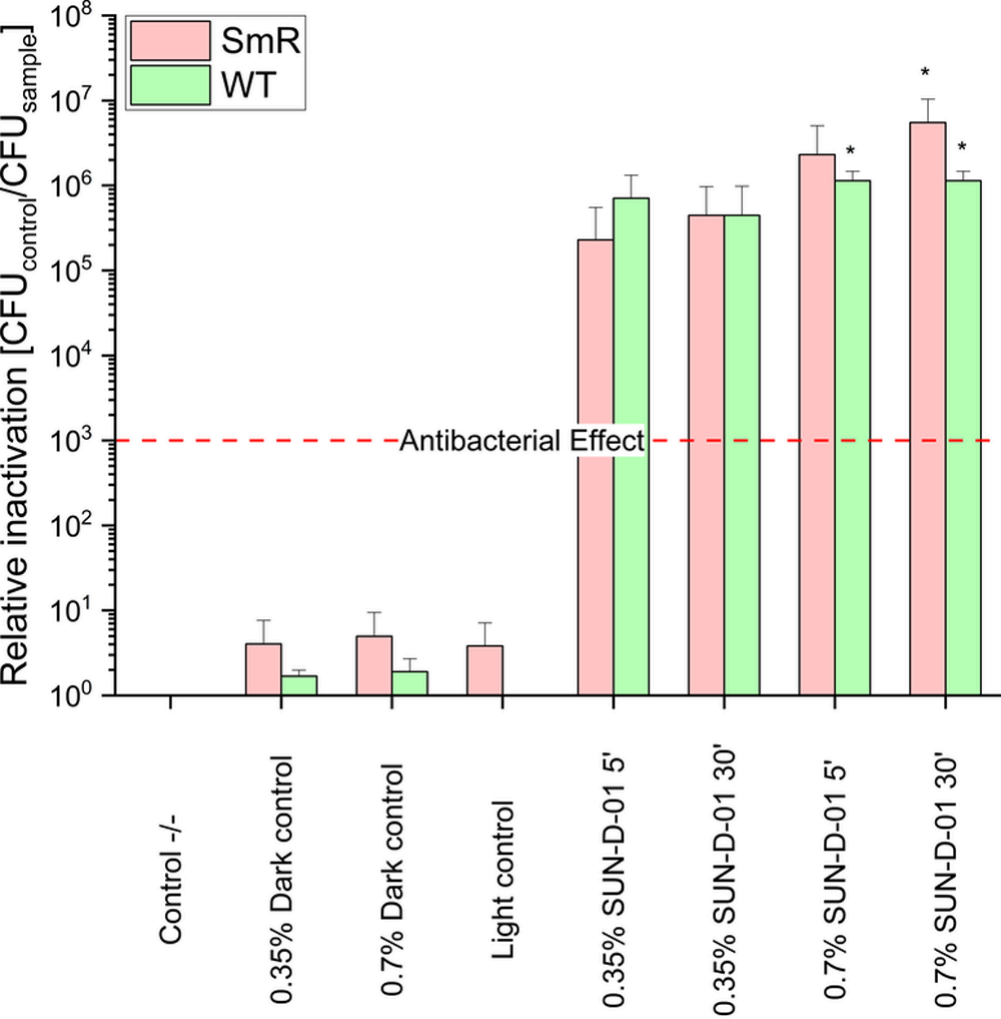
Relative inactivation by photodynamic inactivation of ^WT^ and ^SmR^ cultured in LB medium, using the photosensitizer formulation SUN-D-01 at 0.35 and 0.7% and lower bacterial densities. Each bar reports results from three replicate samples. Incubation: 250 μL, 5 and 30 min, double-sided illumination, 395 nm, 53.2 J cm^–2^, control −/–: double negative control, light control: light only control, no photosensitizer. Number of CFU on control −/–: 1.14 × 10^7^/mL (E.a^WT^), 5.57 × 10^7^/mL (E.a^SmR^). The red dashed line represents the antibacterial effect, corresponding to a reduction of 3 log units. The asterisk * represents a total kill in all replicates. Error bars denote standard deviation. Relative inactivation is CFU control −/– divided by the CFU sample.

Additionally, SUN-D-01 was tested at its standard and doubled concentration when applying the radiant exposure of 53.2 and 78.9 J/cm^2^ and employing a higher cell density than in the above experiments. Figure 5A shows the effect of the SUN-D-01 formulation at the standard concentration of 0.35% against the very high cell density of both strains. The threshold of an antibacterial effect is achieved when applying a 30 min dark incubation and illuminating at 78.9 J/cm^2^. Upon doubling the SUN-D-01 concentration to 0.7% (Figure 5B), the effects are more pronounced. When increasing the radiant exposure by 50% to 78.9 J/cm^2^, an additional phototoxic effect in both strains is observed while maintaining no light toxicity.

**Figure 5:**
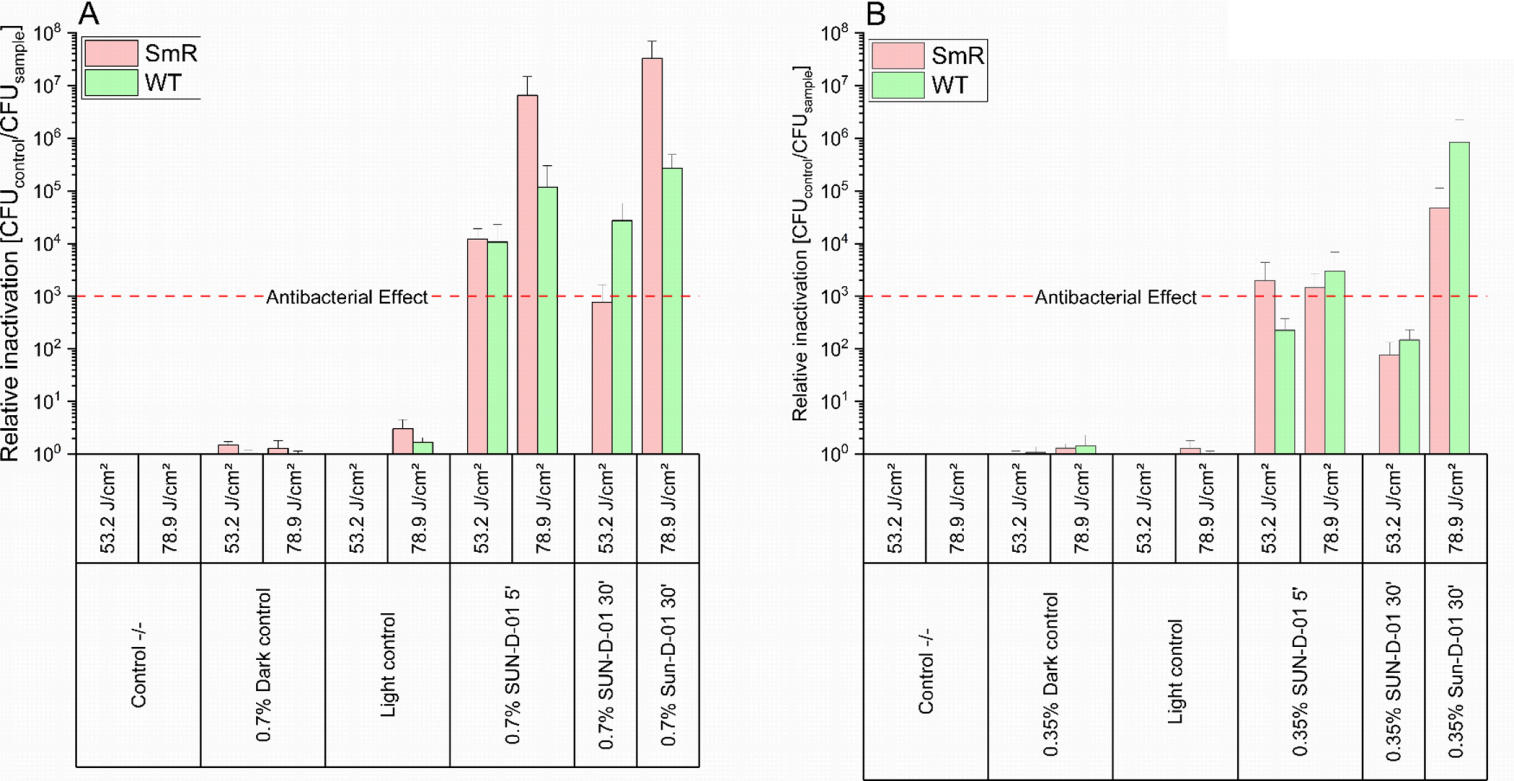
Relative inactivation by photodynamic inactivation of ^WT^ and ^SmR^ cultured in LB medium, using the photosensitizer formulation SUN-D-01 at 0.35 (A) and 0.7% (B) and higher bacterial densities. Each bar reports results from three replicate samples. Incubation: 250 μL, 5 min, double-sided illumination, 395 nm, 53.2 and 78.9 J cm^–2^, control −/–: double negative control, light control: light only control, no photosensitizer. Number of CFU on control −/–: 2.34 × 10^9^/mL (E.a.^WT^ at 53.2 J cm^–2^), 2.78 × 10^8^/mL (E.a.^WT^ at 78.9 J cm^–2^), 3.64 × 10^9^/mL (E.a.^SmR^ at 53.2 J cm^–2^), 4.63 × 10^9^/mL (E.a.^SmR^ at 78.9 J cm^–2^). The red dashed line represents the antibacterial effect, corresponding to a reduction of 3 log units. Error bars denote standard deviation. Relative inactivation is CFU control −/– divided by the CFU sample.

When assessing the efficacy of SUN-D-06, the experiments were conducted employing the photosensitizer formulation at 0.23 and 0.11%, and with and without streptomycin supplementation in the media, to investigate any potential additive effects of the antibiotic in conjunction with PDI.

As depicted in Figure 6, SUN-D-06 at 0.11% and 0.23% both achieved a greater than 3-log steps photo antibacterial effect against ^*WT*^ and ^*SmR*^. The higher concentration of SUN-D-06 (0.23%) showed the highest antimicrobial activity throughout the three sets of experiments. Of the two concentrations studied, it was also the one to consistently provide total bacterial eradication in every replicate. Furthermore, combining PDI with streptomycin did not significantly increase bacterial inactivation of the streptomycin-resistant strain compared to using PDI alone. Taking into consideration these results and the risks that the use of antibiotics represents, as well as the regulations they are subjected to, PDI alone is a preferred method of use for fire blight management.

**Figure 6:**
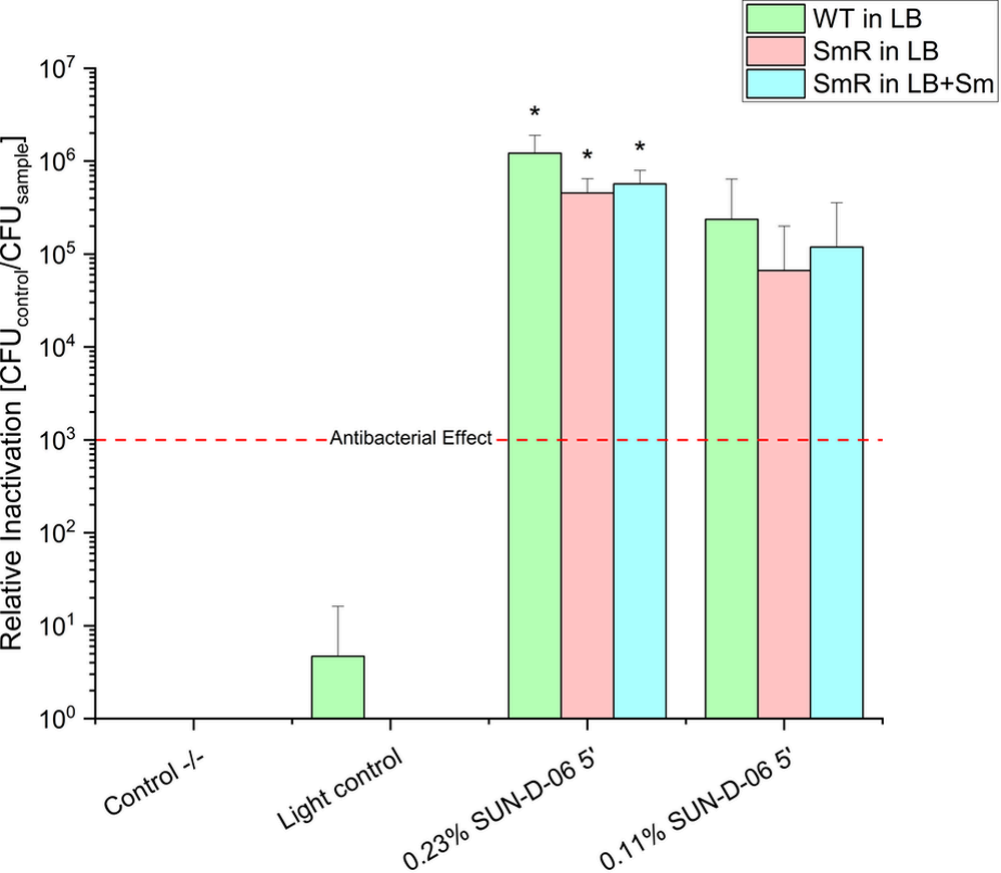
Relative inactivation by photodynamic inactivation of ^WT^ and ^SmR^ cultured in LB medium, and ^SmR^ cultured in LB medium + 100 μg/mL streptomycin, using the photosensitizer formulation SUN-D-06 at 0.23 and 0.11%. Each bar reports results from four replicate samples. Incubation: 250 μL, 5 min, double-sided illumination, 395 nm, 53.2 J cm^–2^, control −/–: double negative control, light control: light only control, no photosensitizer. Number of CFU on control −/–: 8.43 × 10^6^/mL (E.a.^WT^), 5.55 × 10^6^/mL (E.a.^SmR^ in LB), 4.00 × 10^6^/mL (E.a.^SmR^ in LB + 100 μg/mL Sm). The red dashed line represents the antibacterial effect, corresponding to a reduction of 3 log units. The asterisk * represents a total kill in all replicates. Error bars denote standard deviation. Relative inactivation is CFU control −/– divided by the CFU sample.

### 3.3 *^WT^* Resistance Assay to PDI Treatment

To verify that PDI is a safe treatment option for outbreaks in crops and to ensure that it does not induce bacterial resistance with repeated use, we grew the bacteria for 15 PDI cycles, and the surviving CFUs were assessed after each cycle. As Figure 7 shows, a consistent change in relative inactivation was not detected after 15 consecutive PDI treatments. It is noteworthy that the bacteria did not exhibit resistance to PDI using photosensitizer SUN-D-06 at 0.23%. If any alteration was observed, it leaned toward an increased susceptibility, as evidenced by the occurrence of a total kill in every other experiment, starting from the eighth treatment cycle. Each treatment that led to a total kill was repeated, and the resulting number of CFUs fell within the limits of detection, allowing for the study’s continuation.

**Figure 7:**
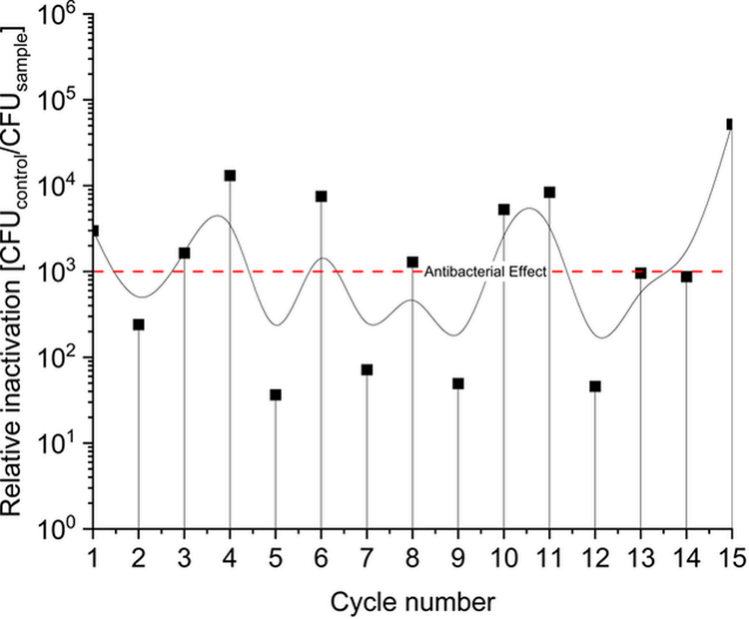
Evaluation of the resistance potential to Photodynamic Inactivation of ^WT^ using the photosensitizer formulation SUN-D-06 at 0.23%. Bacteria were cultured in LB medium and retreated for 15 cycles. Incubation: 250 μL, 5 min, double-sided illumination, 395 nm, 35.11 J/cm*^2^*. Number of CFU on control −/–: 4.96 × 10^6^/mL. The red dashed line represents the antibacterial effect, corresponding to a reduction of 3 log units. The b-spline interpolation connects the data points. Relative inactivation is CFU control −/– divided by the CFU sample.

### 3.4 Mechanism Study with Ce6 and EDTA

#### Uptake of Ce6 with and without EDTA into 

Flow cytometry was conducted to analyze the uptake of Ce6 into to determine how EDTA enhances the photodynamic antibacterial effect of Ce6. The bacterial suspensions (5 × 10^6^ CFU/ml) exhibited a fluorescence intensity of about 34 RFU after being treated with Ce6 alone for 3 h, but 5 mM EDTA enhanced the fluorescence intensity to 70 RFU in 0.5 h and 130 RFU in 3 h (Figure 8A–C). CLSM analysis showed a similar trend (Figure 8D,E). These results indicate that EDTA promoted the uptake of Ce6 by .

**Figure 8:**
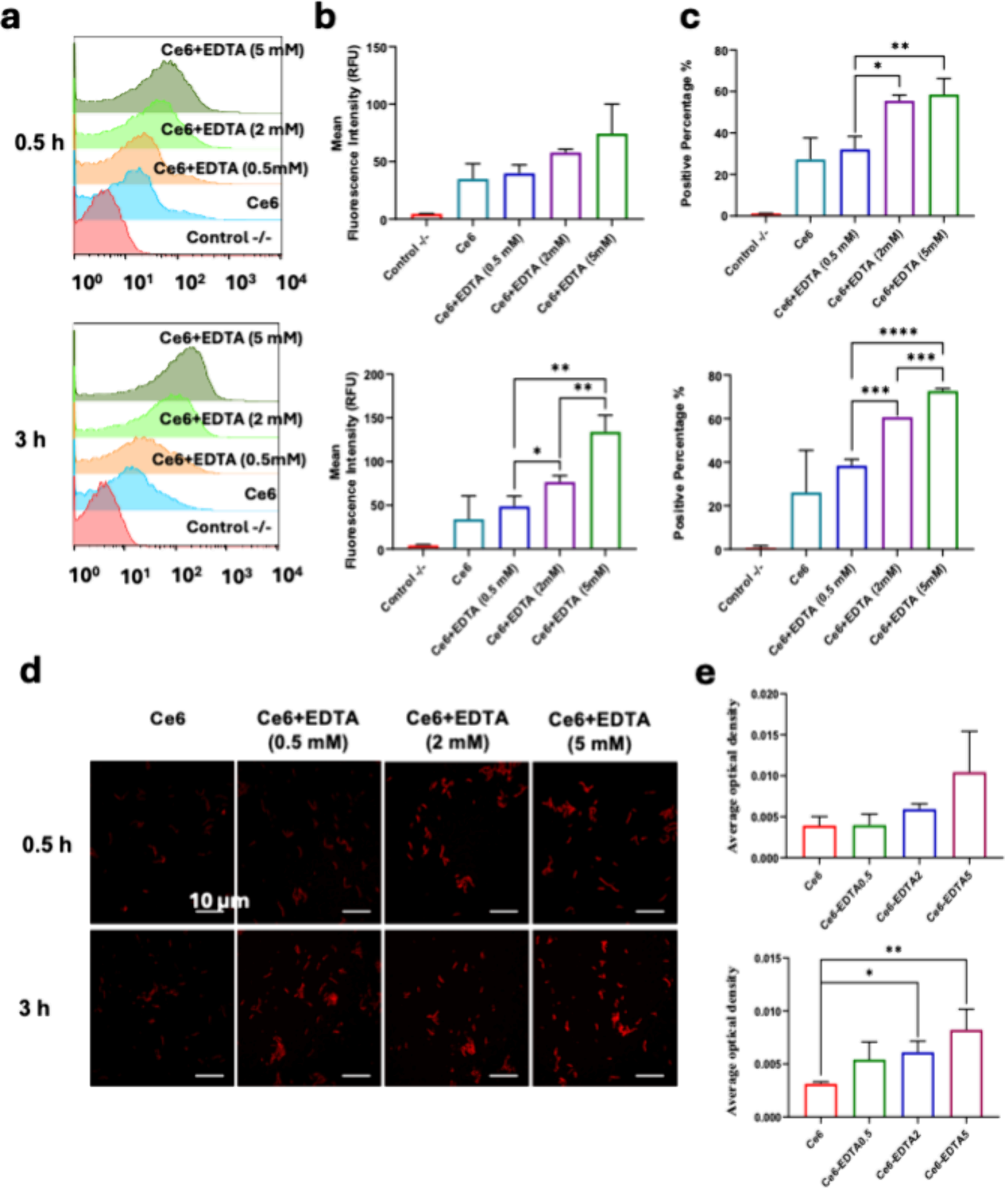
Effect of EDTA on the cellular uptake of chlorin e6 (Ce6) by . (a) Flow cytometry patterns, (b) mean fluorescence intensity, and (c) Ce6-positive percentage of after different treatments. (d) Confocal microscopy imaging of Ce6 in from the Ce6 fluorescence (red signal) at 670–720 nm excited at 640 nm and (e) the integration of the fluorescence intensity analyzed by ImageJ. The bacterial suspension was incubated with Ce6 (0.2 mM) or its combination with different concentrations of EDTA for 0.5 or 3 h. Data are presented as mean ± SD (*n* = 3). ****P < 0.0001, ***P < 0.001, **P < 0.01.

#### ROS Detection of Ce6 with and without EDTA in 

We next studied how EDTA enhanced the antibacterial photodynamic activity of Ce6. It is known that Ce6 generates ROS under irradiation to exert PDT activity. So, the ROS generation in bacteria was probed using dihydroethidium (DHE), which is oxidized by ROS to generate fluorescence at 610 nm. The bacteria treated with Ce6 had a mean fluorescence intensity of 37 RFU, while those treated with Ce6 combined with EDTA (0.27 mM) had an RFU of 60 (Figure 9). However, bacteria treated with EDTA had an RFU of 5, almost the same as the untreated control, suggesting that EDTA promoted Ce6 accumulation in the bacteria and enhanced ROS generation.

**Figure 9:**
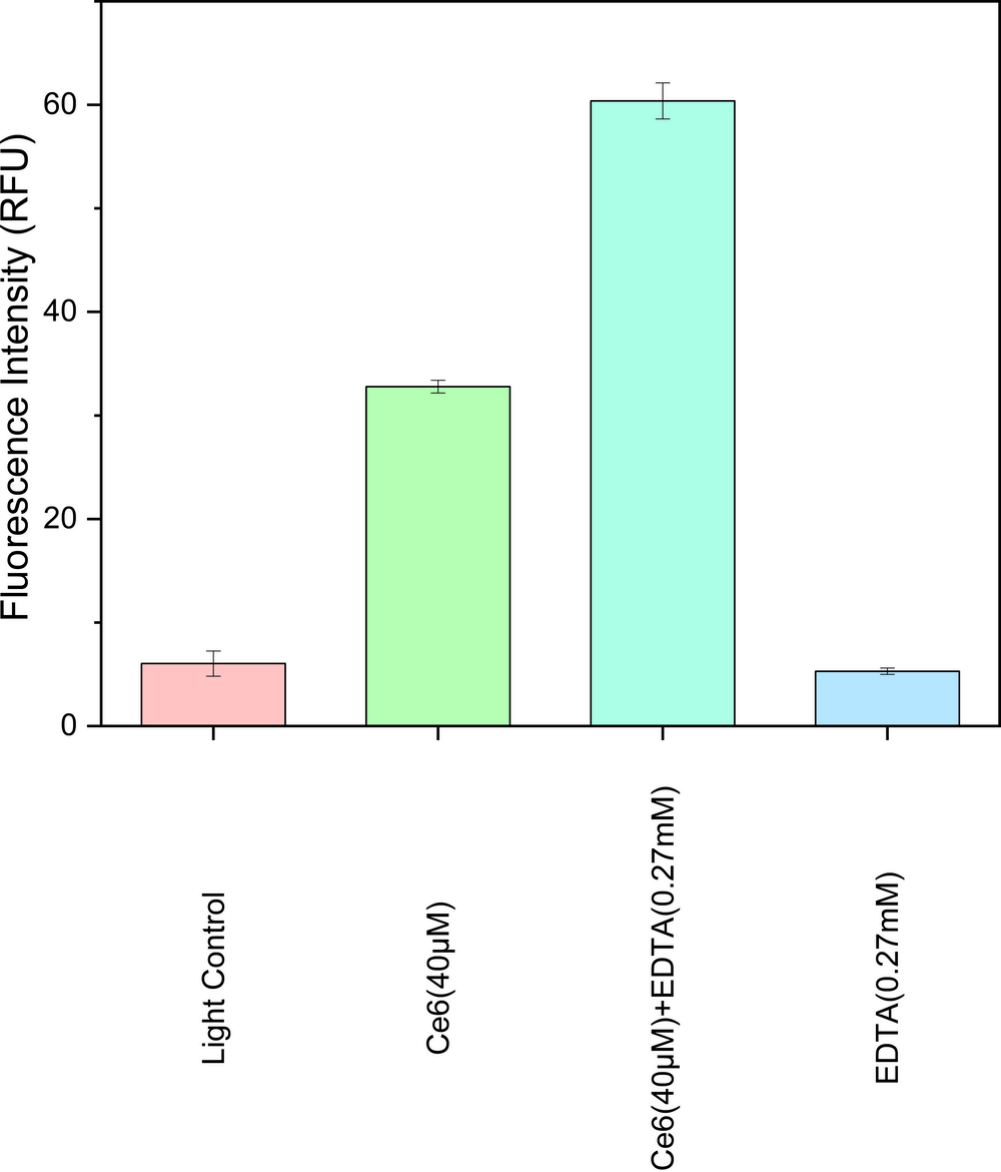
Reactive oxygen species generation of chlorin e6 (Ce6) detected by dihydroethidium (DHE) in . Exponentially growing cells (∼1 × 10^7^ CFU mL^–1^) were preincubated with Ce6 (40 μM) or Ce6 with EDTA (0.27 mM) for 30 min, followed by illumination at 9 mW/cm*^2^* for 1 h (resulting in a radiant exposure of 32.4 J/cm*^2^*). The bacteria were then added with ROS probe-DHE diluted to 1:2000 and incubated for 30 min. The RFU (excitation = 360 nm, emission = 610 nm) was measured by a microplate reader.

#### Modeling of Interaction between EDTA and Ce6 with Gram-Negative Bacterial Membranes

##### Gram-Negative Bacterial Membrane Model

Molecular dynamics (MD) simulations were conducted on a GPU-accelerated workstation utilizing GROMACS Version 2022.3. The consensus within the scientific community affirms the asymmetry of the outer membrane in Gram-negative bacteria, featuring lipopolysaccharides (LPS) on the outer leaflet and a phospholipid bilayer on the inner leaflet.^49,50^ To study the interaction of the membrane’s LPS layer, we prepared an MD model containing 19 LPS molecules on either leaflet generated using the CHARMM-GUI membrane builder (http://charmm-gui.org/),^51^ incorporating the Charmm36 force field.^52^ To make the simulations more efficient, a symmetric structure was created consisting of two leaflets of the bacteria’s outer membrane that do not include the inner leaflet. As the lipopolysaccharide (LPS) is the first point of contact on the outer surface of the bacterial cell, an antimicrobial agent must overcome this protective layer to be effective, either by penetrating it or by impairing the integrity of the LPS. Simulating a symmetric bilayer offers the advantage of doubled statistics during the later-described umbrella sampling, as the molecule is pulled through the same structure twice on the upper and lower leaflet.

The LPS all-atom model was generated using the CHARMM-GUI LPS modeler.^53^ To investigate the biophysical interactions at the outer membrane, we selected a model of the K12 strain was selected. This choice was made because a validated all-atom model for the specific LPS structure of is not currently available. However, is an enterobacterium whose LPS composition and overall polyanionic character are known to be structurally analogous to ’s, sharing key core components.^54,55^ Given that our study aims to understand the fundamental electrostatic and permeation mechanisms involving EDTA and Ce6, the well-established model serves as a robust and satisfactory approximation for the Gram-negative outer membrane in this context.

The molecular structure of the LPS molecule is illustrated in Figure 10. The innermost layer comprises lipid A, followed by the core polysaccharide chain. The outer layer has five units of the K12 O-antigen polysaccharide chain, and the sequence is depicted in Figure 10. Additionally, all-atom models of Ce6 and disodium EDTA were constructed using the CHARMM-GUI ligand modeler.^56^ The membrane and ligands were then combined by using the CHARMM-GUI multicomponent builder.

**Figure 10:**
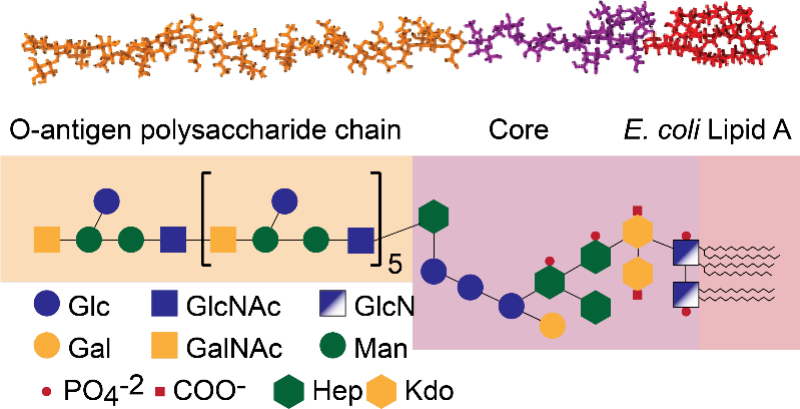
All-atom lipopolysaccharide (LPS) model created by the CHARMM-GUI LPS modeler. The sequence of the O-antigen polysaccharide chain corresponds to that of K12. Abbreviations: glucose (Glc), galactose (Gal), *N*-acetylglucosamine (GlcNAc), *N*-acetylgalactosamine (GalNAc), glucosamine (GlcN), heptose (Hep), mannose (Man), and ketodeoxyoctonic acid (Kdo).

To elucidate the roles of the individual components and their synergy, three distinct MD systems were created by using the symmetrized LPS layer model. This approach allowed us to establish a baseline for Ce6 penetration, characterize the behavior of EDTA alone, and finally study their combined effect. The three systems, termed models 1, 2, and 3, were as follows: model 1 contained an additional single Ce6 molecule and was used to study the interaction between Ce6 and the LPS layer. The interaction of EDTA with the LPS was studied in model 2, which contained an additional single EDTA molecule. Model 3 contained both a single Ce6 and eight EDTA molecules to understand how the presence of EDTA can enhance the LPS layer permeability of Ce6. All systems were charge neutralized and immersed in water containing 0.15 mM NaCl and 0.05 mM CaCl_2_. Ca^2+^ ions are critically important for bacterial cell structure. These cations stabilize the negatively charged LPS outer layer, preventing it from collapsing due to repulsion. The 0.15 mM NaCl concentration is a realistic concentration for the specific environment in which the bacteria live and infect the tree.

All simulations were subjected to a 5 ns minimization and equilibration period in the NPT ensemble (constant pressure and temperature) at 310 K. The simulation employed a 1 fs time step, a short-range van der Waal cutoff of 1.1 nm, and a potential-shift-Verlet Coulomb modifier. Periodic boundary conditions were applied in all spatial directions with neighbor lists updated at intervals of 20 steps. Temperature coupling was regulated by a v-rescale thermostat at a constant pressure of 1 bar, utilizing Parrinello–Rahman semi-isotropic weak coupling (τ = 12 ps; compressibility β = 3 × 10^–4^ bar^–1^). First, the system was allowed to run for 100 ns. Subsequently, the umbrella sampling technique was employed to determine the potential mean force (PMF) along the bilayer normal. In the umbrella sampling technique, a single EDTA molecule (model 1) and a single Ce6 molecule in models 2 and 3 were isolated, and their position was stabilized using a harmonic potential of 1000 kJ mol^–1^. The molecules were then pulled along the bilayer normal at a rate of 0.6 nm/ns. Configurations at increments of 0.2 nm were extracted from this pull trajectory, resulting in 174 configurations. Each configuration is used as the starting point for subsequent biased simulations, referred to as umbrella windows, where the position of the respective molecule remains constrained by the harmonic potential (force constant: 1000 kJ mol^–1^). Each umbrella window was opened for 20 ns. Finally, PMF profiles were generated using the GROMACS built-in weighted histogram analysis method (WHAM).^57^ These PMF profiles were symmetrized between both leaflets.

##### Ce6 and EDTA Interaction with LPS Layer

Umbrella sampling was performed to study the interaction of Ce6 with the LPS layer of Gram-negative bacteria in the presence and absence of disodium EDTA. A bilayer model with LPS on either leaflet was constructed for these simulations, and Ce6 was pulled along the bilayer normal through the bilayer structure in the presence and absence of EDTA.

The LPS layer of Gram-negative bacteria is a pivotal barrier for small molecules.^58^ Understanding how this layer interacts with Ce6 and EDTA is essential for modeling the antimicrobial effects detailed in this article. In Figure 11A, a 3D rendering of the MD model is depicted. The lipophilic core is denoted in gray, the LPS core is colored purple, and the O-antigen polysaccharide sequence is represented in purple and black rods. While the Na^+^ and Ca^2+^ ions are randomly distributed within the water layer, a region with a higher Ca^2+^ concentration in the LPS layer at *z* values of 2 nm < |*z*| < 4 nm has been reported. The concentrated cation layer effectively neutralizes the polyanionic LPS structures, thereby stabilizing the membrane and preventing collapse due to anionic repulsion.^59–61^

**Figure 11:**
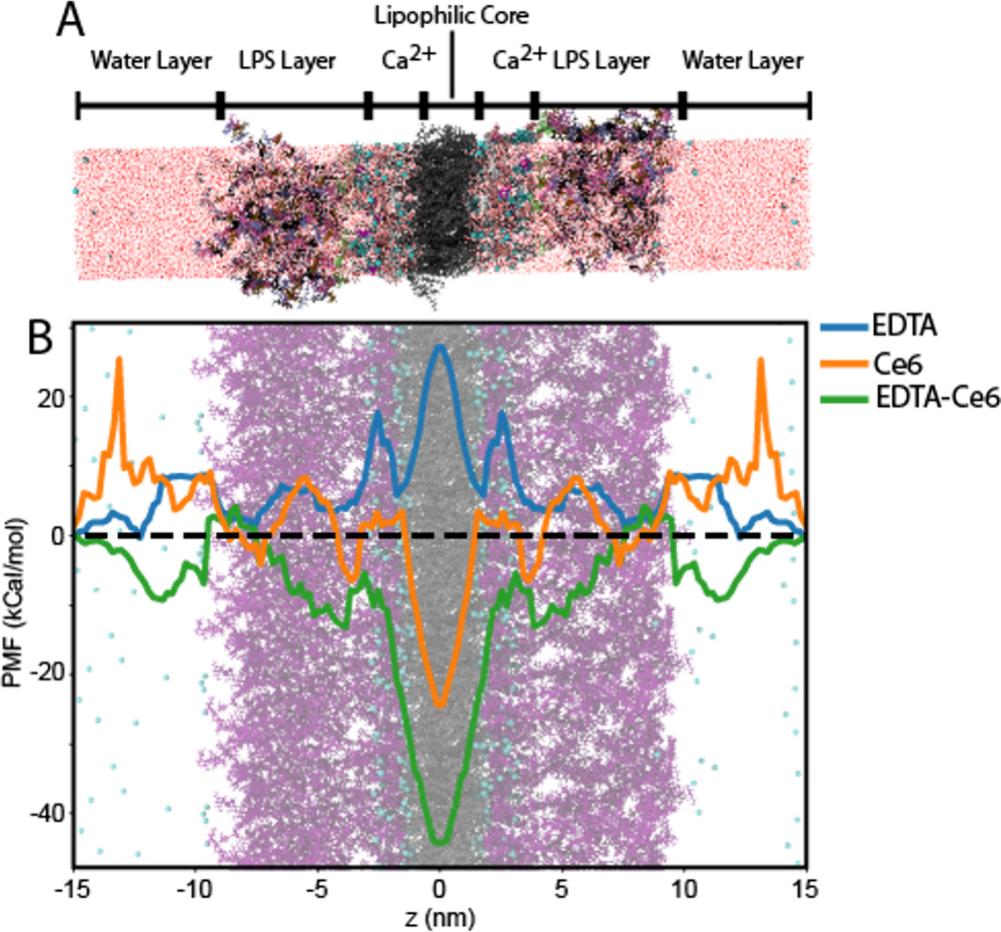
Interaction of chlorin e6 (Ce6) and EDTA with the lipopolysaccharide (LPS) layer of Gram-negative bacterial membranes. (A) A symmetric MD model was designed containing 19 K12 LPS molecules carrying the O6-antigen. The membrane structure consists of the LPS layer and the hydrophobic core of lipid A. The system is immersed in water containing 0.15 mM NaCl and 0.05 mM CaCl_2_. The position of the Ca^2+^ layer within LPS layer is marked. (B) Umbrella sampling was performed on a single EDTA molecule, a single Ce6 molecule and the combination of EDTA and Ce6 across the membrane structure. Ce6 experiences an energy cost of 10 kCAL/mol for penetrating the LPS layer. EDTA has no energy cost for penetrating the LPS layer but a high energy cost for entering the lipid bilayer core region. The energy cost of Ce6 is significantly reduced (green curve at |*z*| ≈ 5 nm) in the presence of EDTA.

The normalized PMF curves are presented in Figure 11B. This PMF profile determines the likelihood of locating a molecule at a specific *z*-position along the bilayer normal in an equilibrated system. A value greater than “0” indicates a barrier, implying reluctance for the molecule to penetrate the structure, while a value of less than “0” suggests a preference for the molecule to reside at that specific location. The PMF profile for EDTA (blue graph in Figure 11B) shows values within ± 10 kcal/mol between the water layer and the LPS layer but increases to approximately 25 kcal/mol in the bilayer center. Conversely, Ce6 exhibits a substantial barrier, reaching a height of 15 kCAL/mol in the LPS core, at |*z*| = 5 nm; however, a pronounced minimum of −20 kcal/mol at the bilayer center (*z* = 0). The introduction of EDTA completely eliminates the energy barrier for Ce6 in the LPS layer and further increases the minimum in the bilayer center to −35 kCAL/mol.

Figure 12A,B depict three-dimensional renders of the MD systems with and without EDTA, highlighting Ca^2+^ and Na^+^ ions as orange and purple beads, respectively. The Ca^2+^ ions are observed either in solution or concentrated at the interface between the lipophilic region and the lipopolysaccharide chains. In the absence of EDTA (Figure 12A), there is a depletion zone of Ca^2+^ and Na^+^ ions within the LPS core in the range of 3 nm < |*z*| < 7 nm. However, when EDTA is present (Figure 12B), both Ca^2+^ and Na^+^ ions are detected within the LPS core. EDTA has a strong affinity for calcium ions and forms stable complexes due to the multiple carboxylic acid groups in its structure. This property of EDTA is widely used in various applications, including in laboratory settings for chelating calcium ions in solutions or as a sequestering agent in water treatment to prevent the formation of scale deposits caused by calcium ions.

**Figure 12:**
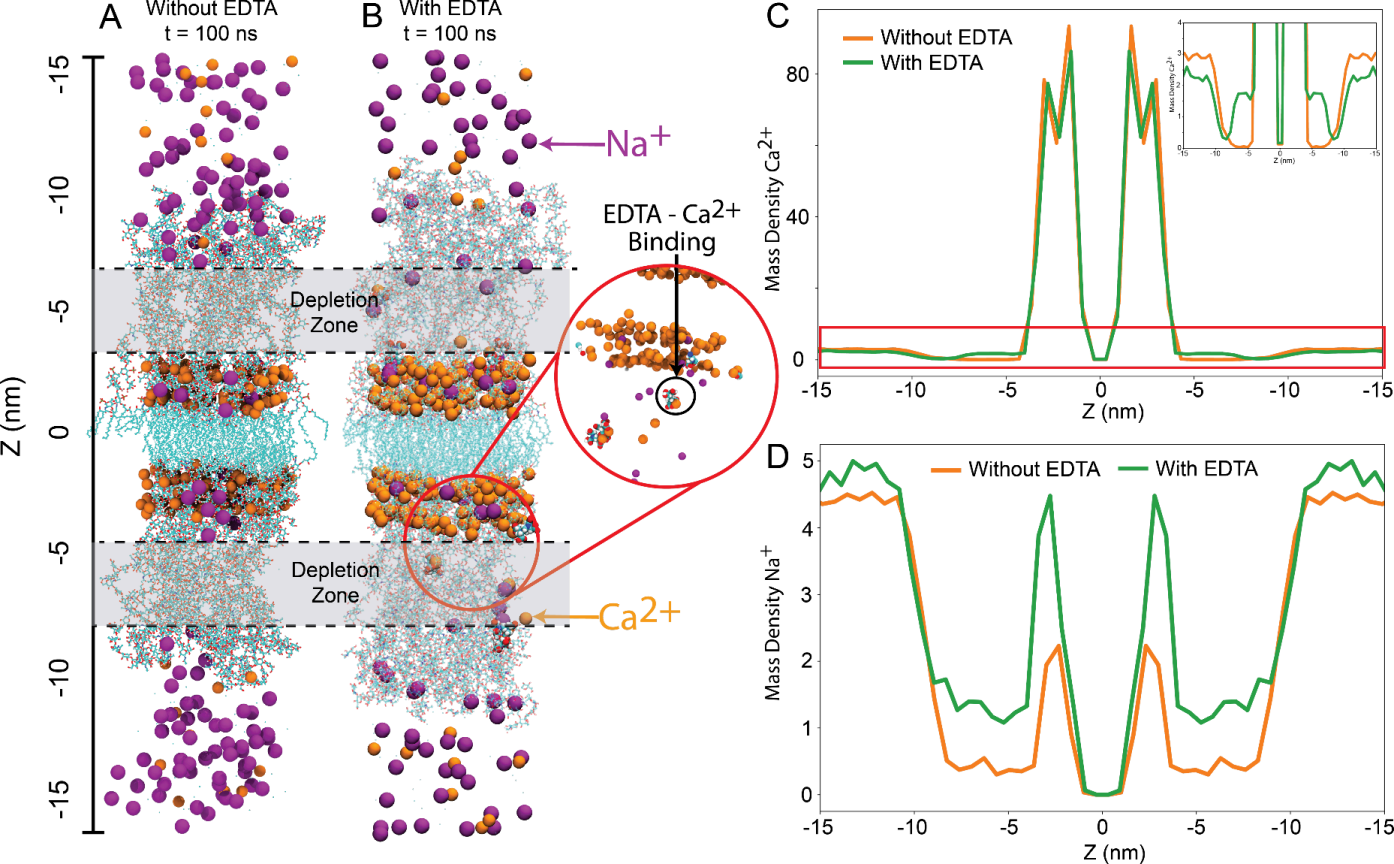
(A) Three-dimensional render of the molecular dynamics (MD) system without EDTA. Ca^2+^ ions are depicted as orange beads, Na^+^ ions are depicted as purple beads, and the lipopolysaccharide (LPS) structure is visualized as turquoise lines. (B) Three-dimensional rendering of the MD system with EDTA. EDTA molecules are depicted as turquoise beads. A zoomed perspective is shown on the right. (C) Mass density of Ca^2+^ along the bilayer normal for the LPS membrane with and without EDTA. The inlet shows the zoomed region marked by the red square. (D) Mass density of Ca^2+^ along the bilayer normal for the LPS membrane with and without EDTA.

The assessment of the ion distribution within the membrane is quantitatively presented through time-average mass density profiles of Ca^2+^ and Na^+^ ions in Figure 12C,D. Figure 12C reveals two prominent peaks, approximately 80 kg/m^3^ each, at |*z*| = 3 nm for Ca^2+^, with the mass density diminishing outside these peaks to values ranging between 0 and 2 kg/m^3^. In the absence of EDTA, the calcium Ca^2+^ mass density measures around 3 kg/m^3^ for |*z*| > 10, progressively reducing to almost 0 kg/m^3^ within the 10 nm > |*z*| > 5 nm range. In contrast, in the presence of EDTA, the calcium Ca^2+^ mass density hovers around 2.5 kg/m^3^ for |*z*| > 10 nm, featuring a peak of approximately 2 kg/m^3^ at |*z*| = 7 nm.

For Na^+^, in the absence of EDTA, the mass density is approximately 5 kg/m^3^ for |*z*| > 10, diminishing to nearly 0.5 kg/m^3^ within the 10 nm > |*z*| > 5 nm range. Notably, the mass density of Na^+^ peaks at 2 kg/m^3^ when |*z*| = 3 nm. In the presence of EDTA, the Na^+^ mass density measures around 4.5 kg/m^3^ for |*z*| > 10 nm, decreasing to almost 1.5 kg/m^3^ within the 10 nm > |*z*| > 5 nm range. At |*z*| = 3 nm, the mass density of Na^+^ is notably elevated, reaching 4 kg/m^3^.

### 3.5 Field trials: *In Vivo* PDI of Fire Blight-Infected Apple Crops

We tested two compounds, SUN-D-01 (2021–2023) and SUN-D-06 (2023), in experiments conducted at the MSU-PLP, and we tested SUN-D-06 in an experiment conducted in Oregon in 2023. In 2021, blossom blight efficacy with SUN-D-01 at each of three different rates was not significantly different from that of the antibiotic standard FireWall (streptomycin), both conferred between 58.9 and 74.6% disease control compared to untreated check trees (Table 4). In 2022, blossom blight efficacy with SUN-D-01 ranged from 68.6 to 80.1% control compared to untreated check trees. In this experiment, the antibiotic standard kasugamycin provided significantly better disease control than the SUN-D-01 treatments, at 95.5% (Table 4). In 2023, SUN-D-01 at 0.36% and SUN-D-06 at 0.12 and 0.24% suppressed blossom blight at a level that was not significantly different from that of the kasugamycin control (Table 4). SUN-D-06 at the lowest rate of 0.06% suppressed blossom blight at a level that was not significantly different than SUN-D-06 at the other rates but was significantly less than that of kasugamycin (Table 4). The test conducted in Oregon relied on natural inoculum and had lower disease pressure (5.3% incidence on the nontreated control trees) than the inoculated tests conducted in Michigan. In this test, SUN-D-06 at all three rates suppressed blossom blight equivalently to kasugamycin and ranged from 71.7 to 84.9% disease control (Table 4).

**Table 4:** Summary of the Efficacy of SUN-D-01 and SUN-D-06 in Reducing the Frequency of the Blossom Blight Phase of Fire Blight at Field Locations in East Lansing, MI, USA, and Harrisburg, or USA from 2021–2023

	application timing^a^					
apple cultivar, year and location of experiment, and treatment	75%	100%	petal fall	blossom blight (%)^b^		blossom blight reduction (%)
Buckeye Gala (2021)	May 4	May 6	May 10			
SUN-D-01 (0.12%)	X	X	X	14.3	bc	59.7
SUN-D-01 (0.24%)	X	X	X	9.0	c	74.6
FireWall (3.3 kg 378 L^–1^)	X	X		10.8	bc	69.6
control				35.5	a	
Buckeye Gala (2022)	May 13	May 14	May 23			
SUN-D-01 (0.12%)	X	X	X	24.5	b	68.6
SUN-D-01 (0.24%)	X	X	X	21.0	b	73.1
SUN-D-01 (0.36%)	X	X	X	15.5	b	80.1
Kasumin (1.9 L 378 L^–1^)	X	X		3.5	c	95.5
control				78.0	a	
Buckeye Gala (2023)	May 9	May 11	May 15			
SUN-D-01 (0.36%)	X	X	X	27.5	bc	57.2
SUN-D-06 (0.06%)	X	X	X	36.0	b	43.9
SUN-D-06 (0.12%)	X	X	X	28.2	bc	56.1
SUN-D-06 (0.24%)	X	X	X	27.2	bc	57.6
Kasumin (1.9 L 378 L^–1^)	X	X		16.2	c	74.8
control				64.2	a	
Braeburn (2023)	Apr 25	May 8	May 20			
SUN-D-06 (0.06%)	X	X	X	1.5	b	71.7
SUN-D-06 (0.12%)	X	X	X	1.5	b	71.7
SUN-D-06 (0.24%)	X	X	X	0.8	b	84.9
Kasumin (1.9 L 378 L^–1^)	X	X		1.0	b	81.1
control				5.3	a	

a Materials were applied to trees at the listed percentage of bloom; dates for stages are listed within rows for each year.

b Values within columns followed by the same letter are not significantly different (*P* < 0.05) according to the LSMEANS procedure with an adjustment for Tukey’s HSD to control for family-wise error.

## 4 Discussion

We here present a novel approach to control fire blight disease caused by by means of PDI based on two PS formulations (SUN-D-01 and SUN-D-06) containing Chl, EDTA and a surfactant. The photoantibacterial strategy presented herein, originally developed for medical applications, has been adapted for use in plant protection through four key approaches aimed at enhancing both environmental sustainability and economic viability: (1) The formulations employed are based on a semisynthetic derivative of chlorophyllin as the active agent, which can be synthesized via a straightforward process and is readily available in large quantities, by this being very economic. (2) The approval of chlorophyllin as food additive E140 within the European Union confirms its high biocompatibility and minimal risk to human health. (3) Upon illumination, Chl undergoes rapid photodegradation, resulting in a self-limiting production of ROS, thereby reducing potential adverse effects on both the host plant and the surrounding environment. (4) Finally, solar radiation serves as the energy source for initiating ROS generation, enabling large-scale treatment without incurring additional energy costs. Our results under high disease pressure in inoculated field trials showed that SUN-D formulations provided consistent efficacy. To the best of our knowledge, this is the first description of such development from the lab bench to the field application.

Our results indicated that Chl in SUN-D-06 acts as a photosensitizer to generate ROS, specifically, singlet oxygen. Sodium magnesium chlorophyllin (Chl) alone was not effective against , even though the strongest treatment option (high radiant exposure, high PS concentration, low bacterial density, and long incubation times) was chosen. This is in line with previous research, showing little toxicity of Chl alone against ,^26,30^ and is evidence that Chl alone would not be effective in combating fire blight. By a combination of Chl with EDTA, SUN-D formulations were effective against the wild-type and the antibiotic-resistant strain of . Decreasing the bacterial density, increasing the radiant exposure, increasing incubation time, and increasing the concentration of the PDI products led to increased photokilling of in our experiments. We argue that the first three of these parameters could cause a better effect in the field, as lower cell densities, higher radiant exposure by sunlight, and longer incubation times would be expected in the field compared to the conditions for laboratory experiments.

The effect of the lower concentration of PDI products in the lab trials could be explained by the better utilization of the available Chl. In the laboratory, we suspect that at higher SUN-D-01 concentrations (0.35 and 0.7%), not enough light reaches the Chl molecules in the center of the 250 μL well to produce sufficient ROS due to strong absorption. For field applications, we expect that even the higher concentrated SUN-D-01 will be doused with enough photons to produce enough ROS to kill the target bacteria, as spraying produces droplets that are far smaller and more spread out than solutions in the well of a 24-well plate.

Despite previous studies showing little bacterial inactivation when chlorophyllin at 1, 10, and 100 μM was combined with EDTA,^26,30^ we demonstrated that the SUN-D formulations, containing Chl, were effective photokilling agents. A hypothesis for this could be the fact that in the present study, the concentration of the photosensitizer was higher, ranging between 0.01 to 0.44 M, and the radiant exposure employed was doubled. Furthermore, an anionic surfactant is also incorporated in the SUN-D formulations, which can enhance the dispersity of photosensitizers and probably alter membrane properties. These three factors combined could explain the favorable outcomes observed with the addition of EDTA in the present study.

When comparing the effectiveness of PDI alone versus PDI combined with streptomycin, it was observed that the combined approach led to a slightly higher level of bacterial inactivation (see Figure 6), consistent with findings by Wimmer et al.^30^ While this synergy between the two methods showed some advantage in antimicrobial efficacy, it is important to consider the financial implications and regulatory concerns associated with antibiotic use. Given the importance of promoting nonresistance-inducing antimicrobial alternatives, it can be concluded that the addition of streptomycin to PDI treatment for ^*SmR*^ may not be necessary. In addition, this study marks the initial investigation into the absence of resistance in after repeated exposure to PDI, and the findings align with the research conducted on and by Bartolomeu^62^ and Pedigo et al.^63^ Although resistance may occur after the 15 cycles studied here, it is still very unlikely that cells are able to develop strategies to protect themselves from the ROS generated by the photosensitizer, since they randomly attack biomolecules such as proteins, lipids, and nucleic acids. Some possible mechanisms have been proposed, but still development of some extent of tolerance, which can be counteracted with either higher concentrations of the photosensitizer or increased radiant exposure, is by far more likely than complete resistance.^64^

To further understand the role of EDTA in the PDI treatments, EDTA was combined with Ce6, the active ingredient of the photosensitizer^65^ in Chl, and the cellular uptake of Ce6 and ROS generation in were compared (Figures 8 and 9). EDTA chelates divalent and trivalent metal ions, such as Ca^2+^ and Fe^3+^,^66^ and thus can sequester metal ions from bacteria. Although EDTA itself has no significant antimicrobial activity, it may potentiate other antimicrobial agents.^67–69^ The EDTA-facilitated intake of Ce6 by and thus high ROS production in the cells may account for this antibacterial activity enhancement by EDTA. As shown in Figure 8, the intake of Ce6 by increased with an increasing EDTA concentration, leading to the production of more ROS in or around the cells upon light irradiation.

Antimicrobial agents targeting the bacterial cell wall or membrane have been effectively used for more than 70 years. The cell wall of a Gram-negative bacterium includes the lipopolysaccharide (LPS) layer, outer membrane, periplasmic space with peptidoglycan layer, and the inner membrane (Figure 13). This complex envelope presents a significant barrier to entry of the drug and is a major determinant of antibiotic resistance. Antimicrobial agents that act against cell walls or membrane targets are among the most commonly prescribed and include penicillin that targets the cell wall and polymyxin B that interacts with the membrane components. However, membrane-active antibiotics must first penetrate the LPS layer before they can interact with the membrane components.^70,71^ In our system, it is known that produces three exopolysaccharides (EPSs),^72^ of which the amylovoran EPS is most important and forms a loose capsule on the exterior of cells.^73^ Despite the presence of the EPS capsule, our demonstration of the requirement of EDTA with Ce6 for cell toxicity coupled with observations of others that indicate that EDTA facilitates the disruption and release of LPS from cells,^57,74^ suggesting that the LPS layer and not the EPS capsule is the barrier to Ce6 uptake by cells.

**Figure 13:**
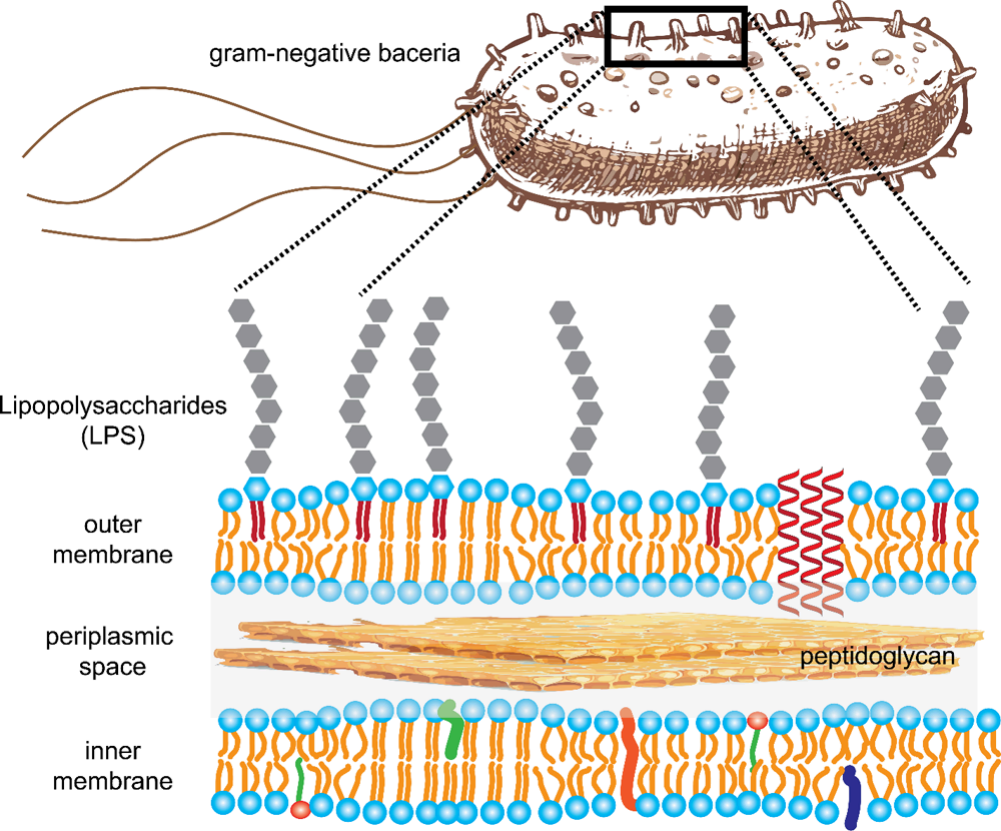
Structural anatomy of a gram-negative bacterial cell wall includes several key components: the protective lipopolysaccharide (LPS) layer, the outer membrane, the periplasmic space with its peptidoglycan layer, and the inner membrane. While past drug development efforts often focused primarily on the membrane components, it is essential to understand all structural elements for comprehensive studies on bacterial pathogenicity, antibiotic resistance, and the development of effective antibacterial treatments. For instance, antibiotics must first penetrate the LPS layer before they can interact with the membrane components.

The computer simulations indicate that the hydrophilic (water-loving) nature of EDTA creates a significant energy barrier, preventing it from entering the membrane’s core. Similarly, the outer LPS layer acts as a physical barrier that blocks Ce6 from reaching the core. Chlorin e6 (Ce6), on the other hand, shows a minimum in the PMF, indicating a preferred location in the membrane core. However, the energy barriers in the LPS layers effectively prevent Ce6 from reaching the core. Our MD simulations propose a two-step mechanism to explain how the combined presence of Ce6 and EDTA leads to the observed antimicrobial effect in our experiments. First, EDTA functions as an LPS active compound that resides in the polysaccharide layer. The presence of EDTA alters the free energy landscape for Ce6, enhancing its likelihood of penetrating the polysaccharide layer and accessing the preferred lipophilic core of the membrane. The PMF profile of Ce6 reveals an energy barrier within the polysaccharide layer. Lipopolysaccharide (LPS) structures, being polyanionic, necessitate charge neutralization, typically facilitated by Ca^2+^ cations. EDTA is well-known for its impact on cation distribution within the LPS layer,^59–61^ and inducing a cascading effect shown in experiments—disrupting membrane stability, elevating permeability, and potentially leading to the collapse of the entire LPS layer due to anionic repulsion.

The analysis of the Ca^2+^ and Na^+^ ion distribution within the system highlights a distinct depleted region in the LPS core when EDTA is absent (Figure 12). The location of the energy barrier impeding Ce6 entry into the LPS layer aligns with this ion-depleted region. The binding of calcium to EDTA plays an important role in actively transporting calcium into the depleted region and influencing the corresponding area. We note that while sodium does not directly bind to EDTA, its levels within the depleted zone also increase in the presence of EDTA. Based on these observations, we suggest that EDTA increases the density of Na^+^ and Ca^2+^ ions in the LPS layer, in particular, in the ion-depleted regions, and that the presence of ions effectively lowers the energy barrier for the amphiphilic Ce6 to penetrate and traverse the LPS layer.

Field testing of SUN-D-01 and SUN-D-06 demonstrated that these formulations were highly effective in suppressing blossom blight on susceptible apple trees with a control equal to that of antibiotic standards in three of four experiments. Management of blossom blight can be extremely difficult because the pathogen can grow exponentially on flower stigmas, readily achieving populations of 10^6^–10^7^ CFU/flower^8^. To date, the antibiotics streptomycin and kasugamycin have been the only compounds capable of rapidly reducing populations on flower stigmas,^75^ and typically provide excellent efficacy that is consistent from year to year.^21,76^ Thus, the efficacy of SUN-D-01 and SUN-D-06 that we observed in replicate field experiments indicates that photodynamic therapy is a highly effective method for fire blight management.

In the field trials, natural sunlight (full spectrum, full day) was used for activation of the photoactive compound. Even though it is difficult to translate the illumination conditions used in the laboratory experiments (LED, 395 nm) to the field experiments, we hypothesize that sunlight reliably activates the photosensitizer due to its long duration and broad spectral coverage. Since effective treatment requires relatively low radiant exposure in the laboratory (typically below 100 J/cm^2^), even moderate sunlight over more than 10 daylight hours provides more than sufficient energy (daily light integral values provided in Table 1). Therefore, we suggest that sunlight is never the limiting factor for the antimicrobial photodynamic effect during the growth period, and successful field trials across different years and under different light conditions confirm that irradiance variations do not impede treatment.

In summary, we introduce photodynamic inactivation as a novel tool for the management of fire blight disease caused by , a Gram-negative bacterial plant pathogen affecting apple and pear trees, as well as other plants from the Rosaceae family. In a set of experiments ranging from *in vitro* laboratory experiments to application in field trials, we demonstrate that PDI represents an effective alternative to the application of antibiotics. Sodium magnesium chlorophyllin serves as an eco-friendly photosensitizer. To be effective against Gram-negative , the addition of EDTA is required to enhance penetration through the bacterial cell wall, which was also proven by molecular dynamic simulations. Therefore, the treatment solutions we developed for field applications, SUN-D-01 and SUN-D-06, contain the photoactive compound Chl, EDTA, and a surfactant. Photodynamic Inactivation is a multifactorial procedure: the photosensitizer concentration, drug-to-light interval, radiant exposure, and the number of bacteria present in the sample determine the photokilling efficacy. Thus, tailoring these parameters for a specific pathogen is mandatory for effective disease control. Once the treatment protocols are established, effective killing of is achieved, irrespective of the resistance status of the target bacterial pathogen against conventional treatment with antibiotics. By this, PDI serves as a treatment even against otherwise resistant bacterial strains. In addition, the procedure does not induce resistance, as proven by repeated treatments. This may be explained by the multitarget attack of the reactive oxygen species toward microbial targets. Our field trials have shown that the PDI-based approach can be applied against fire blight in orchards with very promising efficacy. In conclusion, PDI based on the SUN-D formulations adds a critically needed new tool^77^ to the farmer’s armamentarium against fire blight.

## Supplementary Material



## Notes

Table S1: Eppendorf tubes preparation for the *in vitro* PDI against ^*WT*^ and ^*SmR*^ using Chl and SUN-D formulations; Table S2: data demonstrating the relative inactivation of wild-type and streptomycin-resistant exposed to the photosensitizer SUN-D-06 with and without streptomycin (PDF)

## List of Abbreviations

Ce6: chlorin e6
CFU: colony forming unit(s)
Chl: sodium magnesium chlorophyllin
CLSM: confocal laser scanning microscopy
CMOS: complementary metal-oxide semiconductor
Control – /–: double negative control
DHE: dihydroethidium
DPBS: Dulbecco’s modified phosphate buffer
EDTA: ethylenediaminetetraacetic acid
FCS: fluorescence correlation spectroscopy
Gal: galactose
GalNAc: *N*-acetylgalactosamine
Glc: glucose
GlcN: glucosamine
GlcNAc: *N*-acetylglucosamine
Hep: heptose
Kdo: ketodeoxyoctonic acid
LB: Luria–Bertani
LED: light-emitting diode
LSD: least significant difference
LPS: lipopolysaccharide
Man: mannose
MD: molecular dynamics
MSU-PLP: Michigan State University Plant Pathology farm
NA: nutrient agar
n.a.: not applicable
n.d.: not determined
OD_600_: optical density measured at a 600 nm wavelength
O^2–^: superoxide anions
^1^O_2_: singlet oxygen
PBS: phosphate-buffered saline
PDI: photodynamic inactivation
PDT: photodynamic therapy
PS: photosensitizer
Sm: streptomycin
SOSG: singlet oxygen sensor green
ROS: reactive oxygen species
