# Supplementary material for: Photodynamic Inactivation as a New Weapon Against Plant Fire Blight Disease: Proof of a New Dawn of Environmentally Friendly Crop Protection
Source: J Agric Food Chem. 2025 Aug 1;73(32):19958–73. doi: 10.1021/acs.jafc.5c02074 (PMC12356078; doi:10.1021/acs.jafc.5c02074)
Supplement: Supplementary file 1 [file jf5c02074_si_001.pdf]

Photodynamic inactivation as a new weapon against plant fire blight disease – proof of a new dawn of environmentally friendly crop protection

Mariana Vasconcelos[a], Ying Piao[b], Sebastian Himbert[c], Andreas Fellner[a], Fengyan Wang[d], Jun Liu[e], Michael Fefer[e], Youqing Shen[b], Maikel C. Rheinstädter[c], George W. Sundin[f*], Kristjan Plaetzer[a*]

[a] Laboratory of Photodynamic Inactivation of Microorganisms, Department of Biosciences and Medical Biology, University of Salzburg, Hellbrunner Str. 34, 5020 Salzburg, Austria;
[b] Zhejiang Key Laboratory of Smart BioMaterials and Center for Bionanoengineering, College of Chemical and Biological Engineering, Zhejiang University, Hangzhou 310027, China;
[c] Department of Physics and Astronomy, McMaster University, Hamilton, Ontario L8S 4M1 Canada;
[d] Department of Chemical Engineering, McMaster University, Hamilton, Ontario L8S 4M1 Canada;
[e] Suncor Energy Inc, 150-6 Avenue SW, Calgary, Alberta T2P 3E3, Canada;
[f] Department of Plant, Soil and Microbial Sciences, Michigan State University, 578 Wilson Rd., East Lansing, Michigan 48824, United States

*Corresponding authors: sundin@msu.edu (G.S.) and kristjan.plaetzer@plus.ac.at (K.P.)

Internal – For i

Supporting Information for Publication

Table S 1. Eppendorf tubes preparation for the in-vitro PDI against *Erwinia amylovora*^{WT} and *Erwinia amylovora*^{SmR} using Chl and SUN-D formulations.

Triplets	Sample	ddH2O / PS	Overnight bacterial culture
24-well plate 1	Control -/-	1800 µL ddH_2O	2 µL / 2000 µL ($1.5x10^7$/ $2.72x10^9$ CFU/mL)
24-well plate 1	Light control	1800 µL ddH_2O	2 µL / 2000 µL ($1.5x10^7$/ $2.72x10^9$ CFU/mL)
24-well plate 1	Dark control	1800 µL PS	2 µL / 2000 µL ($1.5x10^7$/ $2.72x10^9$ CFU/mL)
24-well plate 2	200 µM Chl	1800 µL 200 µM Chl	2 µL ($1.5x10^7$ CFU/mL)
24-well plate 2	400 µM Chl	1800 µL 400 µM Chl	2 µL ($1.5x10^7$ CFU/mL)
24-well plate 2	800 µM Chl	1800 µL 800 µM Chl	2 µL ($1.5x10^7$ CFU/mL)
24-well plate 2	0.35% SUN-D-01	1800 µL 0.35% SUN-D-01	2 µL / 2000 µL ($1.5x10^7$/ $2.72x10^9$ CFU/mL)
24-well plate 2	0.7% SUN-D-07	1800 µL 0.7% SUN-D-07	2 µL / 2000 µL ($1.5x10^7$/ $2.72x10^9$ CFU/mL)
24-well plate 2	0.23% SUN-D-06	1800 µL 0.23% SUN-D-06	2 µL ($1.5x10^7$ CFU/mL)
24-well plate 2	0.11% SUN-D-06	1800 µL 0.11% SUN-D-06	2 µL ($1.5x10^7$ CFU/mL)

Internal – For i

Table S 2. Relative Inactivation of *Erwinia amylovora*^{WT} and *Erwinia amylovora*^{SmR} cultured in LB, and *Erwinia amylovora*^{SmR} cultured in LB + 100 µg/mL Sm, using the photosensitizer SUN-D-06 at 0.23% & 0.11%.

Relative Inactivation [$CFU_{control}/CFU_{sample}$]

WT				SmR in LB				SmR in LB+Sm			
Control -/-	hv	SUN-D-06 0.23%	SUN-D-06 0.11%	Control -/-	hv	SUN-D-06 0.23%	SUN-D-06 0.11%	Control -/-	hv	SUN-D-06 0.23%	SUN-D-06 0.11%
1,00E+00	8,53E-01		1,12E+06	1,00E+00	1,98E-01	3,33E+05	3,33E+05	1,00E+00	9,28E-01	5,73E+05	1,98E+01
1,00E+00	5,82E-01		8,56E+00	1,00E+00	1,02E+00	8,40E+05	4,86E+00	1,00E+00	7,81E-01	1,02E+06	1,00E+01
1,00E+00	1,94E-01		1,98E+00	1,00E+00	4,98E-01	3,73E+05	5,21E+00	1,00E+00	3,71E-02	5,79E+04	1,14E+01
1,00E+00	2,45E-01		3,39E+00	1,00E+00	3,02E-01	3,35E+05	1,36E+00	1,00E+00	5,59E-01	6,03E+05	1,26E+01
1,00E+00	8,55E-01		5,39E+05	1,00E+00	5,42E-01	3,95E+05	2,33E+00	1,00E+00	8,88E-01	5,95E+05	5,95E+05
1,00E+00	5,21E-01		5,86E+00								
1,00E+00	9,34E-01		9,28E+02								
1,00E+00	2,86E+00	5,94E+05									
1,00E+00	5,88E-01	5,97E+05									
1,00E+00	3,22E+00	1,47E+06									
1,00E+00	4,07E+01	2,21E+06									
